# Extracting preference relations from data: Clustering with transitive centroids

**DOI:** 10.3758/s13428-025-02674-7

**Published:** 2025-05-07

**Authors:** Debora de Chiusole, Luca Stefanutti, Andrea Brancaccio

**Affiliations:** Department of Philosophy, Sociology, Education and Applied Psychology, University of Padua, Via Venezia, 14, 35131 Padova, Italy

**Keywords:** Pair comparisons, Clustering, Transitivity, *k*-modes, Preference

## Abstract

A clustering algorithm, named *k*-orders, is proposed to extract transitive relations from a data set. The *k*-orders algorithm differs from the original *k*-modes only in the adjustment step. Two adjustment procedures, named transitive centroid adjustment (TCA) and greedy TCA, are proposed that can be used to find clusters with transitive centroids. The proposed clustering approach finds application, especially in studies on preference, where this last may be heterogeneous across individuals, although transitive. The set of cluster centroids extracted by the algorithm from a data set can then be empirically tested via the estimation of a latent class model. The performance of the two versions of *k*-orders were compared to one another and with the canonical *k*-modes, in simulation studies. Results show that when centroids are transitive relations, both versions of *k*-orders outperform *k*-modes. Moreover, in experimental designs in which two-component options are considered, the TCA algorithm performs better than the greedy TCA. An empirical application was also carried out for exemplifying how *k*-orders can be useful for studying individual preferences.

## Introduction

Studying individual preference and, more generally, human choice behavior has always been a research interest of psychology and economy scholars. Some of the more critical issues in this field rely upon the *transitive property* of order relations. The property simply states that given three options *a*, *b*, and *c*, if *a* is preferred to *b*, and *b* is preferred to *c*, then it must be true that *a* is preferred to *c*.

Transitivity of preference is a fundamental aspect of rational choice behavior, and various alternatives have been proposed, for example, the notion of semi-transitivity by Fishburn (1982) and Suzumura consistency (Suzumura, 1976). Moreover, as pointed out by Regenwetter et al. (2011), transitivity of choices might actually be violated less often than it might appear at first glance.

Recently, Regenwetter et al. (2021) published a review summarizing the main issues concerning transitivity and the main procedures for testing it with empirical data. Among the issues considered by Regenwetter et al. (2021), two are central in the present research. The first issue is known, in mathematical theory of voting, as the *Condorcet paradox* (Condorcet, 1785) and it states that a sample composed of subjects with transitive preferences can violate transitivity at the aggregate level. The second issue refers to the modeling of *heterogeneity* of the individual preferences, which is based on the very plausible assumption that different preference relations exist in a group of subjects (see, e.g., Pollak and Wales, 1992).

In the present research, a clustering algorithm is proposed that allows for the extraction of a collection of preference relations, which satisfy transitivity, from preexisting data sets. The extracted preference relations are called “preference states”, and the collection of all the preference states is named “preference structure”.

When the aim is to partition the subjects of a sample into different groups, thinking of a clustering algorithm is quite reasonable. Indeed, this statistical technique should solve the heterogeneity issue described above. Nevertheless, a clustering algorithm is typically based on aggregate statistics like the mode (i.e., *k*-modes algorithm by Chaturvedi et al., 2001), the median (i.e., the *k*-median algorithm by Jain & Dubes, 1988) or the mean (i.e., the *k*-means algorithm by Hartigan & Wong, 1979), that can incur in the Condorcet paradox described above. This contra-position is the reason why clustering techniques are not appropriate for interpreting the cluster centroids in terms of order relations.

A more appropriate approach based on clustering techniques would consist of (i) applying a clustering algorithm to the data for finding the groups of subjects, (ii) applying a probabilistic model, like the Bradley–Terry–Luce model (BTL Bradley & Tarry, 1952) or the Plackett–Luce model (Chapaaan & Staelin, 1982; Plackett, 1975), to each group, and (iii) interpreting the measurement scale values estimated by the model for each group in terms of preference. However, also this approach is limited because the measurement scales obtained for different groups cannot be directly comparable (Swait & Louviere, 1993).

The novelty of the present research is that a *k*-modes clustering algorithm is proposed in which the cluster centroids are constrained to be transitive relations. The rationale behind this approach can be understood by considering the potential drawbacks of the *k*-modes algorithm. Although *k*-modes is highly flexible, this flexibility can sometimes lead to overfitting, particularly when the data are noisy. In fact, in these cases, even when all decision-makers have a transitive preference, *k*-modes might incorrectly identify centroids that are intransitive to best fit the observed data. By adding precise constraints to the algorithm, it may be possible to better capture the true underlying transitive preferences, reducing the risk of overfitting and ensuring more accurate results.

The set of preference states extracted by the algorithm from a data set is then empirically tested by applying a probabilistic model with latent classes to another data set. A good fit of the model to the data would endorse the assumption that centroids are transitive.

The manuscript is organized as follows. “Background and preliminary concepts” section is devoted to (i) a description of the forced-choice paired comparison (FCPC) experiments design, which is one of the typical designs used for preference studies, and to (ii) the presentation of the main characteristics of the classical *k*-modes algorithms. “An algorithm for extracting preference relations from data” section gives a detail description of the new proposal, named *k*-orders algorithm, for extracting preference relations from data. In particular, two alternative algorithms are presented. The performance of both algorithms was compared to one another and with the original *k*-modes in three simulation studies presented in “Simulation Study 1: One component”, “Simulation Study 2: Two component”, and “Simulation Study 3: The selection criterion” sections. The first two studies aimed to evaluate the new algorithms’ capability to extract preference relations from data across various research designs. The third study focused on testing a selection criterion for identifying the best solution, which was subsequently used in the empirical application. The empirical application (”Empirical application: The lockdown social dilemma” section) illustrates how these algorithms can be successfully applied to real data. Specifically, due to the critical situation that humanity has faced in the last years, our attention was focused on the coronavirus (COVID-19) pandemic. More precisely, it was interesting to study individual preferences in different scenarios involving a “lockdown dilemma”. Some final remarks (“Summary and final remarks” section) concludes the argumentation.

## Background and preliminary concepts

### Forced-choice paired comparisons

In FCPC experiments, a single stimulus presents the participant with two options, and the task of the participant is to choose one of them. Different types of options can be considered, which differ for the number of “components” each option is made of. In the base type (subsequently called “one-component” type), a nonempty and finite set *S* of options is considered. A single stimulus in the experiment is represented by an unordered pair $\{s,t\}$ of options in *S*. *Q* represents the collection of all the stimuli. The observed response to a stimulus $\{s,t\}$ (i.e., the choice made by the participant) is represented by the ordered pair (*s*, *t*) if *s* was chosen for that stimulus, and by (*t*, *s*) if *t* was chosen. Given the “forced-choice” nature of the task, no other options are available. This way, the collection of all the participant’s observed responses is a subset *R* of the Cartesian product $S \times S$ and, thus, it forms a binary relation on *S*. Just because of the forced-choice task, if there are no missing responses, and every pair $\{s,t\} \subseteq S$ is presented exactly once, the relation *R* is, by construction, asymmetric (*sRt* implies not *tRs*) and complete (*sRt* or *rSt* holds). This is an assumption that will be carried forward throughout the entire article. An example of one-component options could be found in all those experiments where there is a pair comparison between commodities. An option could be: “You are going to buy a new car. What type of engine would you choose between *Diesel* and *Electric*?”

In the two-component type, options can be formed in a more sophisticated way, described as follows. Let *V* and *W* be two nonempty and finite sets. The set of the options is $S = V \times W$, and, thus, the total number of options forming *S* is $|V| \cdot |W|$. In this case, each stimulus of the experiment consists of an unordered pair $\{(v,w),(v',w')\}$, where both (*v*, *w*) and $(v',w')$ belong to *S*. The observed response of a participant is the ordered pair $((v,w),(v',w'))$ if option (*v*, *w*) is chosen, and $((v',w'),(v,w))$ otherwise. Hence, the collection of all the responses of a participant to the stimuli is a subset *R* of $(V \times W) \times (V \times W)$. Also in this case, for the same reasons explained for the one-component type, the binary relation *R* is asymmetric ($(v,w)R(v',w')$ implies not $(v',w')R(v,w))$ and complete ($(v,w)R(v',w')$ or $(v',w')R(v,w)$). An example of two-component options could be found in all those experiments where the effect of two independent variables on preference is studied. A rather typical one is that where the first independent variable is time and second independent variable is monetary reward. An option could be: “Do you prefer to receive 1000 dollars after 1 year or 500 dollars after 6 months?”

Transitivity occurs in several descriptive theories of decision-making. It simply states that given three options $s_1$, $s_2$, and $s_3$, if $s_1$ is chosen between $s_1$ and $s_2$, and $s_2$ is chosen between $s_2$ and $s_3$, then it must be true that $s_1$ is chosen between $s_1$ and $s_3$. This statement holds true for all triples of options under consideration.

In addition to the transitivity of preferences, when the force-choice pair comparison experiment is based on two-component options, with both components consisting of a linear order of levels, other theoretical assumptions may be required. One such assumption is the so-called *Monotonicity Axiom*. This axiom is defined with respect to a relation *R* on the Cartesian product $S = V \times W$. It is as follows.

(MA): Given any two elements $a,b \in V$ and any two elements $x,y \in W$:
  $$\begin{aligned}&(a,x) R (b,x) \implies (a,y) R (b,y),\\&(a,x) R (a,y) \implies (b,x) R (b,y). \end{aligned}$$

In words, if any two levels of a component are ordered, then this order holds irrespectively of each and every level of the other component.

In the context of this article, an asymmetric and complete binary relation on the set *S* of options is called a *preference relation*. It consists of $$\left( {\begin{array}{c}|S|\\ 2\end{array}}\right) $$ ordered pairs (*s*, *t*) of elements in *S*. More precisely, it contains exactly one ordered pair (*a*, *b*) for each of the $$\left( {\begin{array}{c}|S|\\ 2\end{array}}\right) $$ unordered pairs. Since every single unordered pair is associated with two distinct ordered pairs, by asymmetry, a total number of $$2^{\left( {\begin{array}{c}|S|\\ 2\end{array}}\right) }$$ distinct preference relations are theoretically given. For convenience, each such relation is represented as a binary vector of length $$\left( {\begin{array}{c}|S|\\ 2\end{array}}\right) $$. To give an example, with $|S| = 3$ options, one has $$\left( {\begin{array}{c}3\\ 2\end{array}}\right) =3$$ distinct unordered pairs, and thus, $2^3=8$ distinct preference relations. Table 1 lists all of them as binary vectors (Column 1) and as sets of ordered pairs (Column 2). In this collection, there are exactly two intransitive relations, namely 000 and 111.

**Table 1:** The eight preference relations for the set $A=\{a,b,c\}$ represented as binary vector (column 1) and as sets (column 2)

*Vector representation*	*Set representation*
000	$\{(a,b),(b,c),(c,a)\}$
001	$\{(a,b),(b,c),(a,c)\}$
010	$\{(a,b),(c,b),(c,a)\}$
100	$\{(b,a),(b,c),(c,a)\}$
011	$\{(a,b),(c,b),(a,c)\}$
101	$\{(b,a),(b,c),(a,c)\}$
110	$\{(b,a),(c,b),(c,a)\}$
111	$\{(b,a),(c,b),(a,c)\}$

The only intransitive relations are 000 and 111

### The *k*-modes algorithm

The *k*-modes and *k*-means algorithms are examples of *centroid models* for data clustering. They partition a multidimensional data set into a collection of classes (the *clusters*) by implementing a similarity principle according to which some measure of dissimilarity within the classes is minimized. The cluster centroid is a particular element whose “distance” (dissimilarity measure) from all the other elements in the cluster is minimum. The *k*-modes algorithm was firstly proposed by Huang (1998) as an adaptation to categorical data of the well-known *k*-means (Hartigan & Wong, 1979). Like this last, *k*-modes is an iterative method. It is particularly suitable for multidimensional categorical data, with an arbitrary number of categories for each of the dimensions. Given the qualitative nature of the data, *k*-modes differs from *k*-means for two critical aspects: the dissimilarity measure and the cluster centroid. As for the former, whereas *k*-means uses the Euclidean distance as a dissimilarity measure, *k*-modes uses the Hamming distance. Concerning the latter critical aspect, *k*-means obtains the cluster centroid by computing the element-wise arithmetic mean of the cluster. Instead, in *k*-modes, the cluster centroid is the element-wise mode of it.

The dichotomous (two-category) restriction of *k*-modes, which is the one of interest in this article, is briefly described. The illustration is carried out by using a set-theoretical notation (see e.g., de Chiusole et al., 2017; 2020), which turns out to be convenient in relation to the methods proposed in the present paper. Let *Q* be a set of dichotomous stimuli. The collection of all the responses of an individual to the whole set of stimuli is named a *response pattern* and it is represented by the subset $X \subseteq Q$ of all those stimuli receiving a positive response.

The observed data set is represented by a pair $(\mathcal {R},F)$, where $\mathcal {R}= 2^Q$ is the power set on the set *Q* and $F:\mathcal {R}\rightarrow \mathbb {R}$ is a function assigning observed frequencies to response patterns. In particular, *F* is such that $F(X) \ge 0$ for all $X \in \mathcal {R}$, and $\sum _{X \in \mathcal {R}} F(X) = N$, where *N* is the size of the data set. Given an arbitrary initial collection $\mathcal {C}_1 \subseteq \mathcal {R}$ of “cluster centroids”, the $k$-modes algorithm is carried out in a number $m > 0$ of iterations. Each iteration $i=1,2,\ldots ,m$ consists of two tasks:

- (KM1) given the collection $\mathcal {C}_i$, the *N* observed response patterns are classified into a number of $|\mathcal {C}_i|$ distinct clusters, each represented by a centroid $C \in \mathcal {C}_i$;
- (KM2) each centroid $C \in \mathcal {C}_i$ is adjusted for minimizing the mean discrepancy between *C* and the patterns in the cluster represented by *C*. The collection of the adjusted centroids is $\mathcal {C}_{i+1}$.

The step (KM1) is carried out as follows. A dissimilarity measure between a response pattern $X \in \mathcal {R}$ and a centroid $C \in \mathcal {C}$ is the canonical distance, defined as

$$ |X \Delta C| = |(C \setminus X) \cup (X \setminus C)|, $$

where $X \Delta C$ is the symmetric difference between *X* and *C*.

Given an arbitrary collection $\mathcal {C} \subseteq \mathcal {R}$, the partition of the *N* observed patterns into the $|\mathcal {C}|$ clusters is represented by the *partition function* $f: \mathcal {R}\times \mathcal {C} \rightarrow \mathbb {R}$, which satisfies:

- (C1) $f(X,C) \ge 0$ for all $X \in \mathcal {R}$ and $C \in \mathcal {C}$,
- (C2) $$\sum _{C \in \mathcal {C}} f(X,C) = F(X)$$ for all $$X \in \mathcal {R}$$.

The partition function *f* assigns *f*(*X*, *C*) out of *F*(*X*) occurrences of *X* to the cluster represented by *C*.

A measure of dissimilarity within the cluster represented by a centroid $C \in \mathcal {C}$ is then obtained as a weighted sum of symmetric distances:

$$ D_f(\mathcal {R},C) = \sum _{X \in \mathcal {R}} f(X,C)|X \Delta C|. $$

The goal is to find a partition function *f* that minimizes the overall discrepancy

1 $$\begin{aligned} D_f(\mathcal {R},\mathcal {C}) = \sum _{{C} \in \mathcal {C}}\sum _{X \in \mathcal {R}} f(X,C)|X \Delta C|, \end{aligned}$$

which is the sum of the within-class dissimilarities. For this, the following notation will be used. For $X \in \mathcal {R}$, let

$$ d_{\textrm{min}}(X,\mathcal {C}) = \min _{C \in \mathcal {C}} |X \Delta C| $$

be the minimum distance of response pattern *X* from the collection of centroids $\mathcal {C}$. Furthermore, define the collection $\mathcal {C}_X = \{C \in \mathcal {C}: |X \Delta C| = d_{\textrm{min}}(X,\mathcal {C})\}.$ It is shown in Huang (1998) that the overall discrepancy is minimized if, in each iteration *i* of $k$-modes, the partition function is computed as follows: Given any $X \in \mathcal {R}$ and any $C \in \mathcal {C}$

$$ f(X,C) = {\left\{ \begin{array}{ll} F(X)/|\mathcal {C}_X| & \text {if}\,\, C \in \mathcal {C}_X \\ 0 & \text {if}\,\, C \notin \mathcal {C}_X. \end{array}\right. } $$

A partition function defined this way is named *minimum discrepancy partition function*.

Concerning step (KM2) of the $k$-modes algorithm, namely centroid adjustment, for $i > 0$ let $\mathcal {C}_i$ be the collection of centroids obtained at iteration *i*, $f_i$ be any minimum discrepancy partition function for $\mathcal {C}_i$. A new centroid $C_{i+1}$ is obtained as follows: for each stimulus $q \in Q$, the ratio is computed:

$$ \theta _{C_i,q} = \frac{\sum _{X \in \mathcal {R}_q}f_i(X,C_i)}{\sum _{X' \in \mathcal {R}}f_i(X',C_i)}, $$

where $\mathcal {R}_q = \{X \in \mathcal {R}: q \in X\}$ is the set of all patterns containing *q*. The ratio $\theta _{C_i,q}$ is the proportion of response patterns containing *q*, among all those assigned to the cluster represented by $C_i$.

Then a decision concerning membership of *q* to $C_{i+1}$ is made by using the following rule, henceforth called the *centroid adjustment rule*:

- if $$\theta _{C_i,q} > 1/2$$ then $$q \in C_{i+1}$$,
- if $$\theta _{C_i,q} < 1/2$$ then $$q \notin C_{i+1}$$,
- if $\theta _{C_i,q} = 1/2$ then $q \in C_{i+1}$ with probability 1/2.

The algorithm terminates when the improvement of the overall discrepancy is less than a small tolerance value.

Before closing this section, it is convenient to provide a formal definition of the notion of a “cluster”. Given any $C \in \mathcal {C}$, define the mapping $p_C: \mathcal {R}\rightarrow [0,1]$ such that for every $X \in \mathcal {R}$

$$ p_C(X)=\frac{f(X,C)}{\sum _{X' \in \mathcal {R}} f(X',C)}. $$

Let, moreover, $\mathcal {G}=\{X \in \mathcal {R}: p_C(X)>0\}$ be the collection of all those response patterns having a positive relative frequency in the cluster of *C*. Then, the name *cluster* is assigned to the pair $(\mathcal {G},p_C)$. Sometimes, where understood from the context, the shortcut notation $(\mathcal {G},p)$ will be used in the subsequent sections of the article.

## An algorithm for extracting preference relations from data

In this section, a class of procedures, named *transitive centroid adjustment* (TCA) procedures are described, whose aim is to transform (to “adjust”) a given strict linear order (transitive, asymmetric, and complete binary relation) *L* into another one which is, possibly, at a minimum distance from a given data set, named the “cluster”. The output of the procedure is a strict linear order that constitutes the constrained centroid of the cluster. The term “constrained centroid” stems from the fact that the binary relation is constrained to be a strict linear order and it is at an either local or global minimum distance from the cluster.

It is important to underline that the new algorithms are exactly the same as *k*-modes in the classification step (KM1) and they only differ in the adjustment step (KM2) that is replaced by the TCA procedures.

**Table 2:** Glossary of terms and notation used in the article

Terminology	Notation	Description
Stimulus	$\{a,b\}$	Any unordered pair of options in *S*.
Observed response	(*a*, *b*)	Any ordered pair of options in *S*.
Response pattern	*R*	Any asymmetric and complete relation for *S*.
Response pattern collection	$\mathcal {R}$	The entire set of all theoretically conceivable response patterns.
Preference structure	$\mathcal {P}$	Any nonempty subset of $\mathcal {R}$.
Preference state	*P*	Any asymmetric and complete relation in the preference structure $\mathcal {P}$.
Cluster	$(\mathcal {G},p)$	A pair where $\mathcal {G} \subseteq \mathcal {R}$ and $p:\mathcal {G} \rightarrow [0,1]$.
Centroid	*C*	The centroid of a cluster $(\mathcal {G},p)$, that is an element of $\mathcal {G}$ at minimum discrepancy from all other elements.
Strict linear order	*L*	A transitive, asymmetric, and complete binary relation.
Linear order collection	$\mathcal {L}$	The collection of all the strict linear orders in $\mathcal {R}$.
Average half distance	$d(R,\mathcal {G})$	Weighted average of canonical distances of *R* from $\mathcal {G}$.
Neighborhood	$\mathcal {N}(L)$	The collection of all the linear orders in the neighborhood of *L*.
Marginal proportion	*p*(*a*, *b*)	The marginal proportion of the ordered pair (*a*, *b*) in a cluster.
Free pairs	$\varphi (L)$	The collection of all ordered pairs $(a,b) \in L$ such that $L-(a,b) \in \mathcal {L}^*$.

### Preliminary definitions and theoretical results

In this section, preliminary notations, definitions, and theoretical results are provided, which are necessary for the development of the two TCA procedures. For convenience, the notation used in this section is summarized in Table 2.

We recall that: *R* denotes an observable response pattern, $\mathcal {R}$ is the collection of all the observable response patterns, $\mathcal {P}$ is a preference structure, $(\mathcal {G},p)$ is a cluster, where $\mathcal {G} \subseteq \mathcal {R}$ and $p: \mathcal {G} \rightarrow [0,1]$ . Finally, *C* is the centroid of a cluster.

Given the nonempty and finite set *A* of objects, a *response pattern* for *A* is any asymmetric and complete binary relation $R \subseteq A \times A$. Since the context of the application of the procedures is FCPC, *R* can be regarded as the set of responses of a participant to an FCPC experiment with the options in *A*. Moreover, let $\mathcal {L}\subseteq \mathcal {R}$ be the collection of all the strict linear orders for *A*.

Given a relation $R \in \mathcal {R}$, and a cluster $(\mathcal {G},p)$, the *average half distance* of *R* from the cluster $(\mathcal {G},p)$ is

$$ d(R,\mathcal {G}) = \frac{1}{2}\sum _{R' \in \mathcal {G}} p(R')|R \Delta R'|, $$

where $d(R,\mathcal {G})$ is one half the weighted average of canonical distances of *R* from all response patterns in $\mathcal {G}$. It is worth noticing that since the minimum canonical distance between two distinct relations in $\mathcal {R}$ is 2, the multiplication by 1/2 is for normalizing this minimum distance to 1.

As stated at the beginning of the section, the procedures described below seek to minimize a distance. This objective can be obtained either locally or globally. The definitions of local and global minimum are thus needed. They are both based on the notion of a “neighbor”.

Two strict linear orders $L,L' \in \mathcal {L}$ are *neighbors* if there exists $(a,b) \in L$ such that $(L \setminus \{(a,b)\}) \cup \{(b,a)\} = L'$. It is easily seen that *L* and $L'$ are neighbors if and only if $|L \Delta L'|=2$. The neighborhood of *L* is the collection

$$ \mathcal {N}(L) = \{L' \in \mathcal {L}: |L \Delta L'| = 2\}. $$

As an example, supposing that $\mathcal {L}$ is the collection of all the linear orders on a set of three elements among those provided in Table 1 (i.e., all the relations except for the first and the last one), the *neighborhood* of the relation $L_1=\{(b,a),(b,c),(c,a)\}$, in $\mathcal {L}$, is

$$ \mathcal {N}(L_1) = \{\{(b,a),(c,b),(c,a)\},\{(b,a),(b,c),(a,c)\}\}. $$

It is easily verified that the canonical distance between $L_1$ and each of its neighbors is 2. On the other side, for all the binary relations in $\mathcal {L}$ that are different from $L_1$ and lie outside its neighbor, the canonical distance is strictly greater than 2.

The strict linear order $L \in \mathcal {L}$ is said to be a *local minimum* with respect to the cluster $(\mathcal {G},p)$ if, for every $L' \in \mathcal {N}(L)$, $d(L,\mathcal {G}) \le d(L',\mathcal {G})$. It is a *global minimum* w.r.t. $(\mathcal {G},p)$ if, for all $L' \in \mathcal {L}$, $d(L,\mathcal {G}) \le d(L',\mathcal {G})$.

A binary relation *R* is said to be a *transitive centroid* of a cluster $(\mathcal {G},p)$ if (i) *R* is a strict linear order (i.e., $R \in \mathcal {L}$), and (ii) it is a global minimum with respect to $(\mathcal {G},p)$.

Given a cluster $(\mathcal {G},p)$, and any pair $(a,b) \in A \times A$ with $a \ne b$, define the collections $\mathcal {G}_{ab} = \{G \in \mathcal {G}: (a,b) \in G\}$, and $\bar{\mathcal {G}}_{ab} = \mathcal {G} \setminus \mathcal {G}_{ab}$. We observe that $\bar{\mathcal {G}}_{ab} = \mathcal {G}_{ba}$. Moreover, define the *marginal proportion* of pair (*a*, *b*) in cluster $(\mathcal {G},p)$ as

$$ p(a,b) = \sum _{R \in \mathcal {G}_{ab}} p(R). $$

The pair (*a*, *b*) is said to be *modal* in cluster $(\mathcal {G},p)$ if $p(a,b) \mathcal {G}e 1/2$. It is said to be *non-modal* otherwise. Notice that while *p*(*a*, *b*) is the marginal proportion of the ordered pair (*a*, *b*) in the cluster $(\mathcal {G},p)$, *p*(*b*, *a*) is the marginal proportion of its inverse (*b*, *a*). Moreover, the equality $p(a,b) = 1-p(b,a)$ holds.

The following lemma provides a link between the marginal proportion of a pair (*a*, *b*) and the average half distance. This lemma will be used as an argument in the proofs of several propositions in the next sections. The lemma simply says that the average half distance equals the sum of the marginal proportions of all the ordered pairs (*a*, *b*) that are not in *L*.

#### Lemma 1

Given any strict linear order $L \in \mathcal {L}$, and any cluster $(\mathcal {G},p)$, the following equality holds true:

$$ d(L,\mathcal {G}) = \sum _{(a,b) \in V \setminus L} p(a,b). $$

#### Proof

Let $\mathcal {G}=\{R_1,R_2,\ldots ,R_n\}$, and define the relation $V = \{(a,b) \in A \times A: a \ne b\}$. We observe that any asymmetric and complete binary relation *L* for *A* is such that for any $(a,b) \in V$, the equivalence $(a,b) \in L \iff (b,a) \in V \setminus L$ holds. That is, the complement $V \setminus L$ equals the inverse relation $L^{-1}$. This implies that there is a bijection $f_L:L \rightarrow V \setminus L$ such that $f_L(a,b) = (b,a)$ for all $(a,b) \in L$. Let the pairs in *V* be arbitrarily ordered so that $V = \{v_1,v_2,\ldots ,v_m\}$, where, for each $j = 1,2,\ldots ,m$, $v_j$ represents a pair in *V*. Let *D* be a *n* by *m* binary matrix whose each entry $D_{ij}$ is such that:

$$ D_{ij} = {\left\{ \begin{array}{ll} 1 & \text {if}\,\, v_j \in L \Delta R_i, \\ 0 & \text {otherwise}. \end{array}\right. } $$

Then, it follows at once that $|L \Delta R_i| = \sum _{j=1}^m D_{ij}$ and, hence

$$\begin{aligned} d(L,\mathcal {G})&= \frac{1}{2}\sum _{i=1}^n\sum _{j=1}^m D_{ij}p(R_i) = \frac{1}{2}\sum _{j=1}^m\sum _{i=1}^n D_{ij}p(R_i). \end{aligned}$$

However, for any fixed *j*, if $v_j =(a,b) \in L$, then $\sum _{i=1}^n D_{ij}p(R_i)=\sum _{R \in \bar{\mathcal {G}}_{ab}}p(R)=p(b,a)=p(f_L(a,b))$. If, on the contrary, $v_j = (a,b) \in V \setminus L$, then $\sum _{i=1}^n D_{ij}p(R_i) =\sum _{R \in \mathcal {G}_{ab}}p(R)=p(a,b)$. Thus, one has

$$\begin{aligned} d(L,\mathcal {G})&= \frac{1}{2}\left( \sum _{(a,b) \in L} p(f_L(a,b)) + \sum _{(a,b) \in V \setminus L} p(a,b)\right) , \end{aligned}$$

and because $f_L$ is a bijection between *L* and $V \setminus L = L^{-1}$,

$$\begin{aligned} d(L,\mathcal {G})&= \frac{1}{2}\left( \sum _{(a,b) \in V \setminus L} p(a,b) + \sum _{(a,b) \in V \setminus L} p(a,b)\right) \\&= \sum _{(a,b) \in V \setminus L} p(a,b), \end{aligned}$$

which is the claimed result. $\square$

### A path-finding TCA algorithm

The basic objective of the first TCA procedure is the following: Given an initial and arbitrary strict linear order $L_0 \in \mathcal {L}$, transform $L_0$ into another strict linear order, which is a (local) minimum of $(\mathcal {G},p)$. Sometimes “being a strict linear order” is not a sufficient requirement. For instance, we may be interested in obtaining a strict linear order that also satisfies (MA). Thus, a more general objective is as follows. Indicating with $\mathcal {L}^*$ the collection of all the strict linear orders that satisfy certain additional pre-specified properties or constraints (e.g., (M A)), and given an initial element $L_0 \in \mathcal {L}^*$, the objective is to transform it into another element in $\mathcal {L}^*$ which is a (local) minimum of $(\mathcal {G},p)$.

The procedure accomplishes this in a stepwise manner: In each new step $i>0$, a new “improvement” $L_i$ is obtained by replacing a given pair (*a*, *b*) in $L_{i-1}$ with its inverse (*b*, *a*), such that the following three conditions are jointly met:

- (TC1) $L_i$ is a strict linear order in $\mathcal {L}^*$ ,
- (TC2) $d(L_i,\mathcal {G})$ is strictly less than $d(L_{i-1},\mathcal {G})$, and
- (TC3) the improvement is locally best.

The procedure terminates at step $n > 0$ if $L_n$ can no longer be improved.

Concerning Condition (TC1), since in each step *i*, a pair (*a*, *b*) is replaced by its inverse, and since the initial relation $L_0$ is a strict linear order, asymmetry, and completeness are preserved in every $L_i$. This is not necessarily true for transitivity. For instance, if $L_{i-1} = \{(a,b),(b,c),(a,c)\}$, and $L_i$ is obtained by replacing (*a*, *c*) with its inverse, the resulting relation is still asymmetric and connected, but it fails to be transitive. Indeed, only a special subset of the pairs in $L_{i-1}$ preserves transitivity in this sense. For $R \in \mathcal {R}$ and $(a,b) \in R$, define the operation

$$ R - (a,b) = (R \setminus \{(a,b)\}) \cup \{(b,a)\}. $$

Then, given any strict linear order $L \in \mathcal {L}^*$, a pair $(a,b) \in L$ is said to be *free* if $L -(a,b) \in \mathcal {L}^*$. Thus, a pair (*a*, *b*) is free if the relation obtained from *R*, by replacing (*a*, *b*) with its inverse, is still transitive. It is said to be *constrained* otherwise. Denote by

$$ \varphi (L) = \{(a,b) \in L: L - (a,b) \in \mathcal {L}^*\} $$

the collection of all the free pairs of *L*.

Summarizing, Condition (TC1) is satisfied if and only if, in each step *i*, the strict linear order $L_i$ is obtained by replacing some pair in $\varphi (L_{i-1})$ with its inverse. That is, one must have $L_i = L_{i-1} - (a,b)$ for some $(a,b) \in \varphi (L_{i-1})$.

Pertaining to Condition (TC2), it consists of selecting $(a,b) \in \varphi (L_{i-1})$ so that

2 $$\begin{aligned} d(L_{i-1}-(a,b),\mathcal {G}) < d(L_{i-1},\mathcal {G}). \end{aligned}$$

The following corollary, which follows from Lemma 1, provides the necessary and sufficient condition for (2).

#### Corollary 1

Given the cluster $(\mathcal {G},p)$, a strict linear order $L \in \mathcal {L}^*$, and any pair $(a,b) \in L$, the inequality

$$ d(L-(a,b),\mathcal {G}) < d(L,\mathcal {G}) $$

holds true if and only if $p(a,b) < 1/2$.

#### Proof

By Lemma 1, we have $d(L-(a,b),\mathcal {G}) = (d(L,\mathcal {G})-p(b,a))+p(a,b)$ and hence, the stated inequality is equivalent to $d(L,\mathcal {G})-p(b,a)+p(a,b) < d(L,\mathcal {G})$, that is $d(L,\mathcal {G})+p(a,b) < d(L,\mathcal {G})+p(b,a)$ which holds true iff $p(a,b)<p(b,a)$. Then, the result follows because $p(b,a)=1-p(a,b)$. $\square$

By Corollary 1, at each step $i>0$ of the procedure, Condition (TC2) is satisfied if and only if $L_i= L_{i-1}-(a,b)$ for any pair $(a,b) \in \varphi (L_{i-1})$ such that (*a*, *b*) is non-modal in $(\mathcal {G},p)$.

Finally, Condition (TC3) puts the additional requirement that at step *i*, the selected pair $(a,b) \in \varphi (L_{i-1})$ must be such that for all $(c,d) \in \varphi (L_{i-1})$,

$$ d(L_{i-1}-(a,b),\mathcal {G}) \le d(L_{i-1}-(c,d),\mathcal {G}), $$

that is, the improvement must be the largest possible for that step. A necessary and sufficient condition for (TC2), which is only based on the proportions *p*, is given below.

#### Proposition 1

Given the cluster $(\mathcal {G},p)$, a strict linear order $L \in \mathcal {L}^*$, and any two pairs $(a,b),(c,d) \in L$, the inequality

$$ d(L-(a,b),\mathcal {G}) \le d(L-(c,d),\mathcal {G}) $$

holds true if and only if $p(a,b) \le p(c,d)$.

#### Proof

By Lemma 1, we have that $d(L-(a,b),\mathcal {G}) = d(L,\mathcal {G})-p(b,a)+p(a,b)$ and $d(L-(c,d),\mathcal {G}) = d(L,\mathcal {G})-p(d,c)+p(c,d)$. Hence, the stated inequality is equivalent to $d(L,\mathcal {G})-p(b,a)+p(a,b) \le d(L,\mathcal {G})-p(d,c)+p(c,d)$, which holds iff $p(a,b)-p(b,a) \le p(c,d)-p(d,c)$, which is equivalent to $p(a,b)-(1-p(a,b)) \le p(c,d)-(1-p(c,d))$ and, hence, to $p(a,b) \le p(c,d)$. $\square$

Thus, according to Proposition 1, Condition (TC3) is satisfied by selecting from $\varphi (L_{i-1})$ any pair (*a*, *b*) whose relative frequency *p*(*a*, *b*) is minimum.

On the whole, conditions (TC1), (TC2) and (TC3) are jointly satisfied at step $i>0$ if $L_i = L_{i-1}-(a,b)$ for some $(a,b) \in \varphi (L_{i-1})$ such that $p(a,b) < 1/2$ and $p(a,b) \le p(c,d)$ for all $(c,d) \in \varphi (L_{i-1})$. The procedure terminates at step $n > 0$ if all pairs $(a,b) \in \varphi (L_{n})$ are modal.

It is now possible to show that the outcome of the algorithm is a local minimum.

#### Proposition 2

The relation $L_n$ obtained at the last step of the path-finding TCA algorithm is a local minimum.

#### Proof

Any relation *L* in the neighborhood of $L_n$ is such that $L = L_n - (a,b)$ for some modal pair, hence we have

$$ d(L,\mathcal {G}) = d(L_n,\mathcal {G}) - p(b,a) + p(a,b) < d(L_n,\mathcal {G}). $$

Thus $L_n$ must be a local minimum. $\square$

### A greedy TCA algorithm

Given the set *A* of options, let $V = \{(a,b) \in A \times A: a \ne b\}$ be the set of all pairs of different options, and let $(\mathcal {G},p)$ be a cluster. The second TCA algorithm is based on the notion of an “acyclic relation”. A nonempty subset $G \subseteq V$ is a *cycle* if there are distinct elements $a_1,a_2,\ldots ,a_n$ in *A* such that $G = \{(a_i,a_{i+1}): 1 \le i < n\} + (a_n,a_1)$. A binary relation $R \subseteq V$ is *acyclic* if it does not contain cycles. Let $\mathcal {A}$ denote the collection of all the acyclic relations for *A*.

Instead of being an arbitrary strict linear order, the starting point of the algorithm is any acyclic relation, that is, at the outset $L_0 \in \mathcal {A}$ . A local minimum is then constructed by adding one pair at the time to $L_0$, in a pre-specified order, until no further pairs can be added. It is a “greedy” algorithm because in doing so, it only looks one step ahead. It should be noted that because the empty set is itself an acyclic relation, one can always set $L_0 = \emptyset$, providing a kind of “unconstrained” special case of the starting point of the algorithm. Moreover, since the algorithm is incremental (it never removes pairs), any other legal choice of the starting point $L_0$ can be regarded as a constraint. In fact, whatever belongs to $L_0$ will be carried on to the final solution of the algorithm. To give an example, suppose one wants a relation that respects (MA). Then, it suffices to build $L_0$ as the collection of all and only those pairs that satisfy (MA). It is easily seen that this collection is an acyclic relation.

The greedy TC algorithm operates as follows. Given any acyclic relation $R \in \mathcal {A}$, define the collection

$$ R^\circ = \{(a,b) \in V \setminus R: R \cup \{(a,b)\} \in \mathcal {A}\} $$

of all those pairs (*a*, *b*), each of which, when added to *R*, gives rise to another acyclic relation.

Let $h:V \rightarrow \{1,2,\ldots ,|V|\}$ be an injective mapping, assigning a natural number to each pair, such that for $(a,b),(c,d) \in V$, $h(a,b) < h(c,d)$ implies $p(a,b) \ge p(c,d)$.

At the outset, let $L_0 \in \mathcal {A}$. At every iteration $i>0$ of the greedy algorithm, the new pair $(a,b) \in V$ is considered such that $h(a,b)=i$. Then the new set $L_i$ is constructed by setting

$$ L_i = {\left\{ \begin{array}{ll} L_{i-1} \cup \{(a,b)\} & \text {if}\,\, (a,b) \in L_{i-1}^\circ ,\\ L_{i-1} & \text {otherwise}. \end{array}\right. } $$

The algorithm terminates at step $n=|V|$. The output of the algorithm is the binary relation $L_n$. It is worth noticing that although the starting point is the empty set for all clusters $(\mathcal {G},p)$, the function *p* provides different orders of pairs in different clusters, thus yielding centroids that are not identical.

#### Proposition 3

The binary relation $L_n$ is a strict linear order.

#### Proof

Since $L_n$ is acyclic by construction, it is also asymmetric. To show that it is complete, assume $(a,b),(b,a) \in V \setminus L_n$. Since $(a,b) \notin L_n$, it must be that for $i = h(a,b)$, $L_{i-1}$ is acyclic, and $L_{i-1} \cup \{(a,b)\}$ includes a cycle *G* containing (*a*, *b*). Since $(b,a) \notin L_n$, it must be that for $j = h(b,a)$, $L_j$ is acyclic and $L_j \cup \{(b,a)\}$ includes a cycle $G'$ containing (*b*, *a*). Without loss of generality, assume $i < j$, so that $L_i \subseteq L_j$. Then $G,G' \subseteq L_j$. Since *G* is a cycle, it can be written as $G = \{(a,b),(b,x_1),(x_1,x_2),\ldots ,(x_k,a)\}$. Since $G'$ is a cycle, it can be written as $G' = \{(b,a),(a,y_1),(y_1,y_2),\ldots ,(y_l,b)\}$. Then, we see that the union $G'' = (G \setminus \{(a,b)\}) \cup (G' \setminus \{(b,a)\})$ is itself a cycle, and $G'' \subseteq L_i$, leading to the conclusion that $L_i$ is acyclic, which is a contradiction. Thus either (*a*, *b*) or (*b*, *a*) must belong to $L_n$. Since $L_n$ is acyclic and complete, it must also be transitive. $\square$

#### Proposition 4

The binary relation $L_n$ is a local minimum with respect to the cluster $(\mathcal {G},p)$.

#### Proof

Let $L' \in \mathcal {N}(L_n)$ be any neighbor of $L_n$. Then there is $(a,b) \in L_n$ such that $L' = L_n \setminus \{(a,b)\} \cup \{(b,a)\}$. By contradiction, suppose that $d(L_n,\mathcal {G}) > d(L',\mathcal {G})$, that is, by Lemma 1

$$ \sum _{(u,v) \in L_n} p(v,u) > \sum _{(u'v') \in L'} p(v',u'). $$

Since $L' = L \setminus \{(a,b)\} \cup \{(b,a)\}$, we must have

$$ \sum _{(u,v) \in L_n} p(v,u) > \sum _{(u'v') \in L_n} p(v',u') - p(b,a) + p(a,b), $$

that is, $p(b,a)>p(a,b)$. This implies that $h(b,a) < h(a,b)$, and that the greedy algorithm considers (*b*, *a*) before (*a*, *b*). Therefore, at step $i=h(b,a)$, it had to be true that $(b,a) \notin L_{i-1}^\circ$, for otherwise (*b*, *a*) would belong to $L_n$. Of course, this holds true for every $L \in \mathcal {L}$, which is a superset of $L_{i-1}$. Namely, for every $L \in \mathcal {L}$, $L_{i-1} \subseteq L$ implies $(b,a) \notin L^\circ$. Since $L_{i-1} \subseteq L'$, this must also hold for $L'$. However, this contradicts $(b,a) \in L'$. Thus $d(L_n,\mathcal {G}) \le d(L',\mathcal {G})$ and therefore $L_n$ is a local minimum. $\square$

## Simulation Study 1: One component

The performances of the two versions of *k*-orders (i.e., the TCA and greedy TCA procedures) and the original *k*-modes clustering algorithms were compared in two simulation studies. Several variables, such as sample size, the amount of error in the data, and the cardinality of the preference structure, were manipulated. It is recalled that an asymmetric and complete binary relation on the set *S* of options is called a preference state. Meanwhile, the nonempty collection of all the preference states observable in a population of individuals is called preference structure.

The algorithms’ capability of extracting the actual preference structures was tested in a study with one-component options (as presented in this section) and in study with two-component options (discussed in “Simulation Study 2: Two component” section). In both studies, the aim was to analyze how different conditions affect the performance of the algorithms. Specifically, the research questions were: (1) When data are generated from transitive relations, do *k*-orders algorithms outperform *k*-modes? (2) Under which conditions do TCA and greedy TCA have different performances?

### Data sets generation and simulation design

The number |*S*| of options was 10, leading to $$|Q|=\left( {\begin{array}{c}10\\ 2\end{array}}\right) =45$$ stimuli. Four variables were manipulated in this simulation design: sample size (i.e., the total number of simulated responses), cardinality of the preference structure, amount of error in the data, and the number of the initial set of centroids.

Sample sizes were 50 and 500, representing “small” and “large” samples, respectively. Three different preference structures were considered: $\mathcal {P}_{10}$ with a cardinality of 10, $\mathcal {P}_{50}$ with a cardinality of 50, and $\mathcal {P}_{100}$ with a cardinality of 100. In the rest of this Section and in the next one, we will refer to $\mathcal {P}_c$ to indicate the preference structure with cardinality $c \in \{10, 50, 100\}$. Each preference structure was obtained by leveraging the bijective correspondence between the set of all permutations of *S* and the family of all strict linear orders on *S*. Specifically, *c* random permutations of the integers from 1 to $|S| = 10$, were generated for each preference structure. Each permutation, in its natural order, was then transformed into the corresponding preference relation *P*.

To give an example of how each permutation was transformed into a preference relation, let us assume that the number of options is $|S|=4$. Then, the permutation (1, 3, 2, 4) is mapped to the preference relation *P* such that $s_1 P s_3 P s_2 P s_4$. The collection of the preference relations *P* obtained this way formed the preference structure $\mathcal {P}_c$.

We expected that preference structures with fewer states might be more accurately reconstructed. In fact, for any constant number of options, as the number of states in the preference structures increases, it becomes more likely that some of them might be similar and difficult to distinguish.

With the aim of generating a simulated dataset, a probability distribution $\pi _{c}$ was assumed on $\mathcal {P}_{c}$. In particular, it was assumed that $\pi _{c}$ was the uniform distribution so that the probability of observing each preference relation $P \in \mathcal {P}_{c}$ was $1/|\mathcal {P}_{c}|$. Thereafter, a certain number *N* of pairs (*R*, *P*), where *R* is a response pattern and *P* is a preference state, were generated. In each pair, *P* was sampled with replacement from the collection $\mathcal {P}_{c}$ by using $\pi _{c}$ as the multinomial distribution, whereas *R* was obtained through the introduction of some amount of error, according to the following rule: For each pair of options $a,b \in S$, if *aPb* then *bRa* with probability $\beta _{ab}$ and *aRb* with probability $1-\beta _{ab}$. This rule just says that if the pair (*a*, *b*) is in *P*, then an inversion of it occurs with probability $\beta _{ab}$.

Five different intervals $\{(0,.10],(.10,20],(.20,.30],(.30,.40],(.40,50]\}$ plus a no-error condition were considered for establishing amount of error in the data. Each interval’s boundaries indicated the lower and upper bounds of the probability $\beta _{ab}$. For each error condition and each stimulus, the values of the $\beta _{ab}$ and $\beta _{ba}$ were randomly generated within the specified interval and kept constant throughout the simulation study. A comprehensive list of all parameters used can be found in the online supplementary material^1^

In summary, a total of 36 conditions were considered by combining three preference structures, two sample sizes, and six error intervals. For each condition, 100 simulated samples were generated, all sharing the same preference structures, sample sizes and error intervals. The final 3600 samples were then used to extract a preference structure using the standard *k*-modes algorithm and the two versions of *k*-orders. The initial sets of centroids were set to have either the same cardinality as the true preference structure, or twice the cardinality of the true preference structure. In both cases, these initial sets were generated at random by extracting them from the power set $2^{Q}$.

### Methods

For each cardinality *c* of the true preference structures, the performances of the three algorithms were compared to one another by using three indexes. The former is a true-positive rate and the other two are variants of a discrepancy measure. All three indexes compare the true preference structure $\mathcal {P}_{c}$ with the preference structure $\hat{\mathcal {P}}_{c}$ extracted by a particular algorithm.

The *true-positive rate* (TPR) was computed as the proportion of true states $P \in \mathcal {P}_{c}$ that belongs to the extracted structure $\hat{\mathcal {P}}_{c}$, that is:

$$ TPR =\frac{|\hat{\mathcal {P}}_{c} \cap \mathcal {P}_{c}|}{|\hat{\mathcal {P}}_{c}|}. $$

The discrepancy measure is the *average minimum discrepancy* between two collections $\mathcal {A}$ and $\mathcal {B}$, computed as follows:

3 $$\begin{aligned} \delta (\mathcal {A},\mathcal {B})=\frac{1}{|\mathcal {A}|} \sum _{A \in \mathcal {A}} d_{min}(A,\mathcal {B}), \end{aligned}$$

where, $d_{min}(A,\mathcal {B})=min_{B \in \mathcal {B}} |A \Delta B|$. The average minimum discrepancy $\delta (\mathcal {A},\mathcal {B})$ varies from 0 to the total number |*A*| of stimuli. In particular, it is 0 when $\mathcal {A}\subseteq \mathcal {B}$. It is worth noticing that this discrepancy is not symmetric, because $\delta (\mathcal {A},\mathcal {B}) \ne \delta (\mathcal {B},\mathcal {A})$ in general. The two indexes $\delta (\hat{\mathcal {P}}_{c},\mathcal {P}_{c})$ and $\delta (\mathcal {P}_{c},\hat{\mathcal {P}}_{c})$ were computed by using the Formula in (3).

In each condition of the study, average values (and the corresponding standard deviations) of both indexes were computed across the 100 simulated samples. Then, the performance indexes computed on the solutions of the three algorithms were compared to one another.

### Results and discussion

Results obtained by using sample size $N=500$ and by setting the initial sets of centroids to be of the same cardinality of the true preference structure are shown. The results of the other conditions (i.e., $N=50$, and cardinality of the initial set of centroids is two times that of the true preference structure) are provided as supplementary material.

**Fig. 1:**
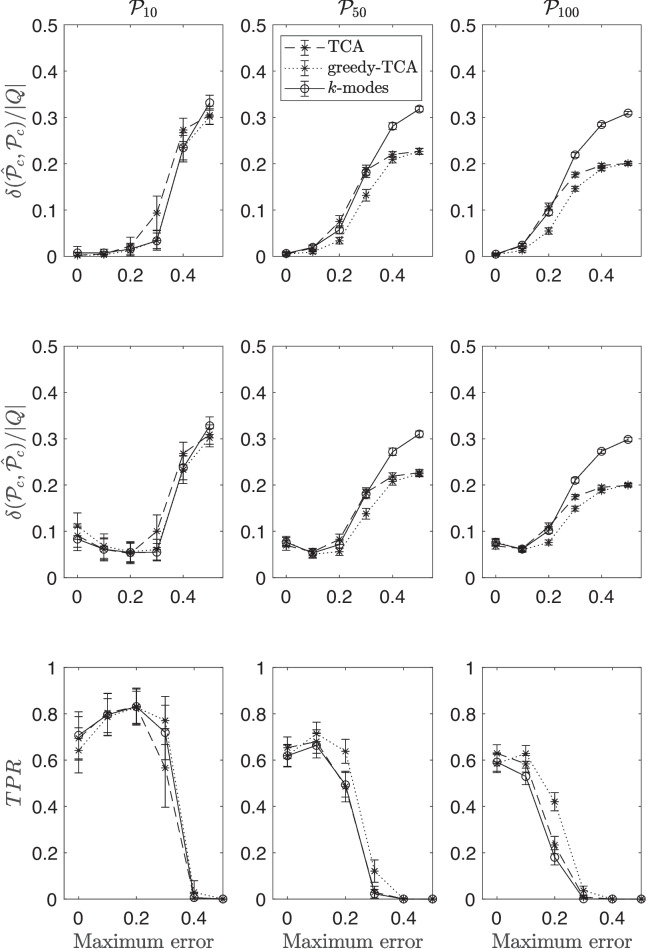
Comparison between the performances of *k*-modes (*straight lines*), the TCA (*dashed lines*), and the greedy TCA (*dotted lines*) procedures, in Simulation Study 1. See text for more details

Figure 1 displays the performance of the three algorithms in all conditions. Top, middle, and bottom panels in the figure refer to $\delta (\hat{\mathcal {P}}_{c},\mathcal {P}_{c})$, $\delta (\mathcal {P}_{c},\hat{\mathcal {P}}_{c})$, and TPR performance indexes, respectively, whereas the left, center, and right panels report the results obtained for cardinalities $c=10$, $c=50$, and $c=100$, respectively. Each panel of the figure shows the results of a particular performance index as a function of the maximum amount of error in the data. Straight lines refer to *k*-modes, dashed lines to TCA, and dotted lines to greedy TCA.

With the exception of the preference structure $\mathcal {P}_{10}$, all the performance indexes suggest that the solutions of the two versions of *k*-orders algorithms are always superior or, equal to that of *k*-modes. The biggest difference in the performance of *k*-modes compared to those of the other two algorithms is in the sensitivity to the amount of error in the data and to the number of preference states. When the true preference structure is $\mathcal {P}_{10}$, a clear superiority of one algorithm over the others does not arise. When it is $\mathcal {P}_{50}$ or $\mathcal {P}_{100}$, the TPR of all algorithms decreases rapidly when the amount of error increases (bottom panels). However, the average minimum discrepancies of the two *k*-orders increase less than that of *k*-modes, remaining relatively small (i.e., around 20% when maximum error is .50). Answering research question (1), results suggest that the two *k*-order algorithms outperform *k*-modes when preference is transitive.

Concerning research question (2), the greedy TCA procedure compared to the TCA displays a slight superiority in conditions where $\mathcal {P}_{50}$ and $\mathcal {P}_{100}$. Indeed, when the amount of error is between .10 and .40, solutions found by the greedy TCA reached the smallest minimum discrepancy and the highest TPR.

Thus, simulation results suggest that when the experiment design is of type one-component options, the greedy TCA should be used for extracting preference states from the data. While these results are promising, it is important to note that the limited number of preference structures considered in this simulation study means the generalizability of the findings is still uncertain and may require further studies.

Concerning the manipulation of the sample size and the cardinality of the initial set of centroids, the results suggest, not surprisingly, that: (i) increasing the sample size, the performance of the clustering algorithms also increases; (ii) Increasing the number of the initial set of centroids, the performance of the algorithms slightly increases, but only when the amount of error in the data is small (i.e., less than .10) . All these results can be verified by looking at the figures reported as supplementary material.

## Simulation Study 2: Two component

Introducing two-component options adds a further constraint to the *k*-orders (i.e., the TCA and greedy TCA procedures) algorithms. Indeed, in two-component tasks, research objectives and theoretical assumptions may require the (MA) to hold in the extracted preference states. In this simulation study, the performance of *k*-orders is compared with that of the original *k*-modes algorithm, using the same variables manipulated in Simulation Study 1 (“Data sets generation and simulation design” section) and the same performance indexes (“Methods” section). Also the research questions remain the same: (1) When data are generated from transitive relations, do *k*-orders algorithms outperform *k*-modes? (2) Under which conditions TCA and greedy TCA have different performances?

### Data sets generation and simulation design

A set *S* of two-component options, defined on $4 \times 4 = 16$ distinct options, was considered. Consequently, the total number of stimuli was $$|Q| = \left( {\begin{array}{c}16\\ 2\end{array}}\right) = 120$$. As in the previous simulation study, sample sizes were manipulated under two conditions, with $N = 50$ and $N = 500$, respectively. The cardinality *c* of the preference structure used to simulate the data was set to 10, 50, or 100.

Given the two components of the options, the procedure for generating the preference structure $\mathcal {P}_c$ differs from that used in Simulation Study 1. Indeed, it is important to note that the stimuli in this type of experiment can be categorized into two sets: those containing “monotone” stimuli and those containing “non-monotone” stimuli. Let $(V, \prec _V)$ and $(W,\prec _W)$ be two linearly ordered sets representing the two components of an experiment. To be precise, each of the two orders $\prec _V$ and $\prec _W$ is a reflexive, transitive, antisymmetric, and complete binary relation. Each stimulus consists of an unordered pair $\{(v_i,w_l),(v_m,w_p)\}$ of options $(v_i,w_l),(v_m,w_p) \in V \times W$. A stimulus $\{(v_i,w_l),(v_m,w_p)\}$ is *monotone* if

4 $$\begin{aligned} v_i \prec _V v_m \implies w_l \prec _W w_p, \end{aligned}$$

and it is *non-monotone* otherwise. Since the left-hand option of the stimulus is less than the right-hand one in both components, it should be expected that preference has the same direction in both components.

Let $P_0 \subseteq (V \times W) \times (V \times W)$ be a binary relation such that given $(v_i,w_l),(v_m,w_p) \in V \times W$,

$$ (v_i,w_l)P_0(v_m,w_p) \iff {v_i \prec _V v_m \text { and } w_l \prec _W w_p}. $$

This relation can be easily shown to be a partial order, known in the literature on order relations as the pointwise order (or *coordinate-wise order*, Davey & Priestley, 2002). The relation $P_0$ provides the starting point for the generation of “random preference relations”, which are complete, asymmetric, and transitive relations generated at random that satisfy the monotonicity axiom. Precisely, each single random preference relation *P* was obtained through the following algorithm: at the outset, $P \leftarrow P_0$, and the collection *I* is built of all the pairs of options in *S* that are incomparable with respect to *P*. Then the following steps are repeated until *I* is empty:

1. among all pairs $(i,j) \in I$ such that $P \cup \{(i,j)\}$ is transitive, one is chosen at random;
2. let $(i',j')$ be the chosen pair. A new version of *P* is obtained by setting $P \leftarrow P \cup \{(i',j')\}$;
3. finally, both $(i',j')$ and $(j',i')$ are removed from *I*.

The relation *P* obtained this way is a strict linear order that satisfies the monotonicity axiom. A preference structure $\mathcal {P}_c$ is then obtained by taking a number $c \in \{10, 50, 100\}$ of random preference relations.

Having available the preference structure, the simulated data sets are obtained with the same procedure described in “Data sets generation and simulation design” section. Five different intervals $\{(0,.10],(.10,20],(.20,$ $.30], (.30,.40],(.40,50]\}$ plus a no-error condition are used in this study for $\beta _{ab}$. For each error condition and each stimulus, the values of the $\beta _{ab}$ and $\beta _{ba}$ were randomly generated within the specified interval and kept constant throughout the simulation study. A comprehensive list of all parameters used can be found in the online supplementary material.

The total number of conditions was 36, obtained by combining three preference structures, two sample sizes, and six error intervals. In this simulation, as in the previous one, 100 different samples were generated, and preference structures were extracted using the standard *k*-modes algorithm and the two versions of *k*-orders. The initial sets of centroids were either matched to the cardinality of the true preference structure or set to twice that size. In both cases, these sets were randomly generated from the power set $2^{Q}$.

A remark concerning two-component design is worthwhile. A critical point is that *k*-order clustering must also respect the monotonicity axiom, meaning that in the final solution of the clustering procedure, every cluster centroid, besides being a transitive relation, must also include $P_0$. Indeed, the two *k*-orders procedures need no particular modification for this.

In both of them it suffices to set $p((v_i,w_l),(v_m,w_p))=1$ for all $((v_i,w_l),(v_m,w_p)) \in P_0$. In fact, in each step of the TCA procedure, a pair is replaced by its inverse only if it is non-modal. Obviously, pairs in $P_0$ are always modal. Hence, they can never be replaced, whereas in greedy TCA, pairs $((v_i,w_l),(v_m,w_p))$ are considered in decreasing order of the proportion $p((v_i,w_l),(v_m,w_p))$. This is equivalent to setting the initial relation equal to $P_0$ rather than $\emptyset$. Since $P_0$ is a partial order, it also respects the requirement that in every single step the relation is acyclic.

### Methods

In each simulation condition, the three performance indexes TPR, $\delta (\hat{\mathcal {P}}_{c},\mathcal {P}_{c})$, and $\delta (\mathcal {P}_{c},\hat{\mathcal {P}}_{c})$ described in “Methods” section were computed to compare the preference structures $\hat{\mathcal {P}}_{c}$ extracted by the algorithms with the true preference structure $\mathcal {P}_{c}$ used to simulate the data. In each condition of the study, average values (and the corresponding standard deviations) of all indexes were computed across the 100 simulated samples. Then, the values obtained were compared to one another.

### Results and discussion

Results obtained by using sample size $N=500$ and by setting the initial sets of centroids to be of the same cardinality of the true preference structure are shown. The results of the other conditions (i.e., $N=50$, and cardinality of the initial set of centroids is two times that of the true preference structure) are provided as supplementary material^2^

**Fig. 2:**
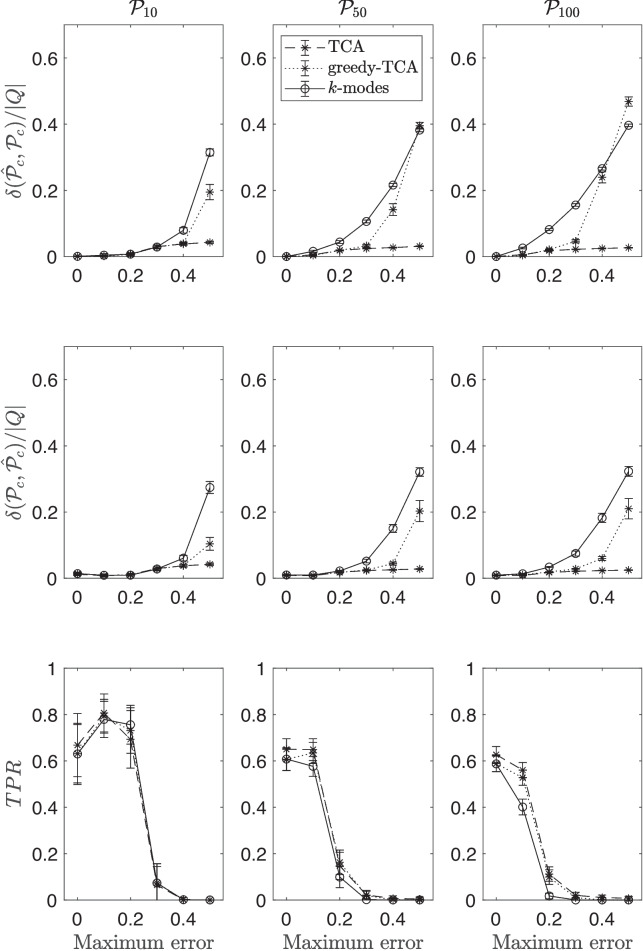
Comparison between the performances of *k*-modes (straight lines), the TCA (*dashed lines*), and the greedy TCA (*dotted lines*) procedures, in Simulation Study 2. See text for more details

Figure 2 displays the results obtained on the performance indexes. It is read like Fig. 1.

All the performance indexes suggest that the solution of both versions of *k*-orders is always better or, at most, equal to that of *k*-modes. This is true irrespective of the number of preference states and of the amount of error in the data. Moreover, it is quite evident that *k*-modes is more sensitive to noise. Indeed, when the amount of error in the data increases, all indexes worsen much slower for TCA and greedy TCA algorithms than for *k*-modes. This strongly suggests that *k*-orders algorithms are better suited when choice behavior is expected to be transitive. These results provide a positive answer to research question (1).

A direct comparison between the TCA and the greedy TCA have shown that the former performs better (answer to research question (2)). Indeed, the average minimum discrepancies obtained for the solutions of TCA remain very close to 0, even when the amount of error in the data is very high (i.e., .50).

Results of this simulation study suggest that when the experiment design is with two-component options the TCA version of the algorithm should be used.

Concerning the manipulation of the sample size and the cardinality of the initial set of centroids, the results suggest, as in the other study, that: (i) increasing the sample size, the performance of the clustering algorithms also increases. (ii) Increasing the number of the initial set of centroids, the performance of the algorithms slightly increases, but only when the amount of error in the data is small (e.g., less than .10) . All these results can be verified by looking at the figures reported as supplementary material.

## Simulation Study 3: The selection criterion

A crucial element in the functioning of clustering algorithms is the initial set of centroids used to start the extraction process. The choice of these centroids significantly influences the performance and accuracy of the algorithms, as they serve as the starting points for the iterative process of forming clusters. Typically, clustering algorithms iteratively consider many different starting points and then select the best solution. To this aim, having a reliable selection criterion is crucial.

This simulation study aims to test a procedure, used in “Empirical application: The lockdown social dilemma” section, that is an adaptation of the *k*-states procedure (de Chiusole et al., 2017), that incorporates a selection criterion proposed by de Chiusole et al. (2020). The procedure, whenever applied to empirical data, is based on a cross-validation technique that partitions the data set into two subsets of response patterns: a training set (two-thirds of the sample) and a validation set (the remaining third). The training set is used to extract the structure, while the validation set is used to test it. In this simulation study, the validation set is not used, since the data were generated from the true preference structure. This partitioning process is repeated 100 times. Each time, the algorithm is set with a number of initial centroids chosen from 1 to 100. The value of 100 initial centroids was chosen as a conservative estimate, set to double the cardinality of the largest true preference structure employed in this simulation study. A “best structure” is then selected using a criterion based on a distance computed between the training set and the extracted structures. The structure with the smallest maximum distance is chosen as the best.

### Simulation design and data set generation

Data sets of an FCPC experiment with two-component options of four levels were simulated. The number of options was $4 \times 4=16$, leading to a number of $$\left( {\begin{array}{c}16\\ 2\end{array}}\right) =120$$ stimuli.

Data sets were generated under two levels of “amount of error” and two “true” preference structures varying in cardinality. The preference structure cardinalities were either 10 or 50. The maximum amount of error in the data was set to .05 (small error) or .30 (medium-high error). For both conditions and each comparison, the values of the $\beta _{ab}$ and $\beta _{ba}$ were randomly generated between zero and the maximum error level of the condition and kept constant throughout the simulation study. A comprehensive list of all parameters used can be found in the online supplementary material.

The sample size was fixed at 400 in all conditions. This value was chosen because it is of the same order of magnitude as the sample size of the empirical study (see “Empirical application: The lockdown social dilemma” section). In each of the $2 \times 2=4$ conditions, 100 data sets were simulated.

### Methods

In each of the four simulation conditions, an adaptation of the *k*-states procedure proposed by de Chiusole et al. (2017) was applied to each of the 100 simulated data sets, resulting in one best knowledge structures for each data set. The procedure consists of three main steps. The first step performs a partition of the data set into a training set and a validation set. The second step, referred to as the “incremental extensions step”, generates a sequence of locally optimal preference structures of increasing size. This process begins with the smallest preference structure (i.e., with cardinality one) and adds a new preference state at each new iteration. A preference structure is extracted, at each iteration, using the *k*-orders algorithm applied to the training set. In the last step, the procedure proposed by de Chiusol et al. (2020, p. 517) was applied to select the “best preference structure”.

Then, in each simulation condition and for each sample, the three performance indexes *TPR*, $\delta (\hat{\mathcal {P}},\mathcal {P})$, and $\delta (\mathcal {P},\hat{\mathcal {P}})$, as described in “Methods” section, were computed to compare the preference structure $\hat{\mathcal {P}}$ extracted by the algorithm with the true structure $\mathcal {P}$. Additionally, the cardinality of the extracted preference structure was considered.

An average value across the 100 samples was then computed for each performance index, in each of the four simulation conditions.

### Results

Table 3 shows the results obtained for each of the performance indexes. As expected, varying levels of error in the data (.05 or .30) and differences in the cardinality of true preference structures (10 or 50) significantly impact the performance of the *k*-order algorithm. In Condition C1, where the error is small, the algorithm performs with high accuracy, achieving a TPR of .95. In the few instances where *k*-order does not extract the true preference states (5% of cases), the discrepancies between the extracted and true structures are minimal (at most one stimulus out of the 120 monotone and non-monotone stimuli).

**Table 3:** Average performance indexes obtained in Simulation Study 3

Condition	$|\mathcal {P}|$	Error	TPR	$\delta (\hat{\mathcal {P}},\mathcal {P})$	$\delta (\mathcal {P},\hat{\mathcal {P}})$	$|\hat{\mathcal {P}}|$
C1	10	.05	.95	0.15	1.06	23.41
C2	10	.30	.74	0.44	2.87	44.59
C3	50	.05	.93	0.17	0.72	50.89
C4	50	.30	.37	1.10	2.39	87.99

The first three columns indicate the condition number, the cardinality of the true preference structure, and the maximum amount of error in the data used for data generation. Columns 4 to 7 display the average values of the performance indexes *TPR*, $\delta (\hat{\mathcal {P}},\mathcal {P})$, $\delta (\mathcal {P},\hat{\mathcal {P}})$, and the cardinality of the extracted preference structure, respectively

In Condition C2, where the amount of error in the data is high, the performance of *k*-order remains quite good. TPR decreases to .74, and the discrepancies between the extracted and true structures stay relatively low, averaging about 3. This suggests that even with a medium-high amount of error, the solutions extracted by *k*-order are still close to the true preference structure.

Similar results were obtained in Conditions C3 and C4, but with lower values for all the performance indexes.

Despite the positive results regarding the quality of the solutions found, there is a notable issue with the cardinality $|\hat{\mathcal {P}}|$ of the extracted structures. Even with a small error level (Condition C1), the cardinality of the extracted structure is more than double (23 instead of 10). When the error level is high (Condition C2), the cardinality is four times greater than expected (45 instead of 10). Interestingly, when the true preference structure is large (i.e., 50 states), the results are significantly better. With a small amount of error, *k*-order extracts structures with nearly the same cardinality of the true one (approximately 51). However, when the error level is medium-high, the algorithm extracts structures with about 50% more states.

## Empirical application: The lockdown social dilemma

This empirical application exemplifies one of the contexts where *k*-order algorithms can effectively address specific research questions about individual preferences. As elaborated in the following subsection, this serves as a proof of concept for the applicability of the algorithm with real-world data.

Due to the critical situation that humanity has faced in recent years, our attention has been focused on the coronavirus (COVID-19) pandemic. More precisely, it could be interesting to study individual preferences in different scenarios involving a “lockdown social dilemma”.

Lockdown measures are all the confinement measures (i.e., curfew) adopted by governments to reduce the contagion curves of the COVID-19 disease. Two central themes of the “lockdown” have been the subject of various political and ethical issues: *economy* and *health*. It is precisely to these aspects that the lockdown dilemma is linked. It can be illustrated by the following two scenarios: “slowing down the economy to save lives” or “sacrifice human lives to avoid economic collapse of a nation”? Some studies (Aum et al., 2021; Kochanczyk et al., 2012) tried to identify a potential compromise between the two aforementioned aspects, arriving, however, at the conclusion that saving lives and protecting the economy are goals that need to be maximized jointly. Thus, the compromise between economy and health interests can be viewed as a dilemma, and, in particular, a social dilemma. The term dilemma refers to the need of making a choice between two options in contrast to each other, without the possibility of choosing a third optimal alternative.

The main dilemmas studied in psychology are ethical, social, and moral. What differentiates a social dilemma from an ethical or moral one is the requirement to choose between two equally complex opposite alternatives, making a decision to favor oneself by causing damage to the community or, vice versa, to make a cooperative choice while neglecting one’s personal gain.

### Aims and scopes

This empirical application is aimed at illustrating how the *k*-order algorithm can be fruitfully applied for learning the preference states in a population when there is no specific a priori knowledge about them. In this respect, the psychological surrounding of the “lockdown social dilemma” was selected because it is expected to provide heterogeneous preferences among individuals. In particular, it is expected that individuals can be partitioned into those who prefer safeguarding health and those who prefer safeguarding the economy. Additionally, in this context, the preference states are assumed to be transitive, resulting in linear orders.

The lockdown social dilemma was chosen because it allowed for the manipulation of the social context, either public or private, in which the options were defined. The rationale behind this decision was that an individual’s choice to prioritize health or the economy could depend on how much their personal interests were threatened. For example, if public health (e.g., the number of seriously ill individuals in a nation) is compared to personal family income, some individuals might prefer to safeguard the economy. Conversely, if private health (e.g., the health of family members) is compared to the public economy (e.g., the number of workers who lose their jobs in a nation), individuals might prioritize health. It is noteworthy that the data of this study were collected during the COVID-19 pandemic between 2020 and 2021. This aspect is particularly important, given the type of options used in the FCPC task.

For these reasons, the lockdown social dilemma presents a particularly challenging context to test the new *k*-modes algorithm.

### Research design, options, and tools description

Four different scenarios were considered, each of which was designed with two-component options. The two components were *economy* and *health*. In each scenario, both components were linearly ordered sets of four levels. They are denoted by $(E_c, \prec _{E_c})$ and $(H_c, \prec _{H_c})$, respectively, where $c \in \{\text {private},\text {public}\}$ is the social context used for contextualizing the options. More in detail, in the options, the two components were referred to as social contexts in which *public* or *private* interests were involved. Thus, a 2 (components) $\times$ 2 (social contexts) factorial design was used, resulting in four contexts.

The variables considered for contextualizing the options in the four contexts were: “Decrease of personal income” for the private economy $E_{\text {private}}$ context, “Percentage of national unemployment” for the public economy $E_{\text {public}}$ context, “Severity of the COVID-19 infection of a family members” for the private health $H_{\text {private}}$ context, and “Number of deaths due to COVID-19 infection” for the public health $H_{\text {public}}$ context.

Four levels of increasing severity were defined for each of the four contexts described above. Thus, the total number of options used for composing the stimuli was 4 (contexts) $\times$ 4 (levels)$=16$. Table 4 lists the 16 options distinguished in the four contexts.

**Table 4:** Description of the 4 (contexts) $\times$ 4 (levels) $=16$ options used for building the stimuli of the empirical application

	Options in private economy $E_{\text {private}}$ context
1	Your family income remains unchanged
2	Your family income decreases by 5%
3	Your family income decreases by 10%
4	Your family income decreases by 40%
	Options in public economy $E_{\text {public}}$ context
1	Today, 1000 people lose their jobs
2	Today 5000 people lose their jobs
3	Today, 10,000 people lose their jobs
4	Today, 20,000 people lose their jobs
	Options in private health $H_{\text {private}}$ context
1	None of your family members dies of COVID-19
2	One of your family members is affected by COVID-19
3	One of your family members is hospitalized due to COVID-19
4	One of your family members dies of COVID-19
	Options in public health $H_{\text {public}}$ context
1	The number of deaths from COVID-19 remains unchanged
2	Compared to yesterday, 100 more new deaths
3	Compared to yesterday, 200 more new deaths
4	Compared to yesterday, 500 more new deaths

The four contexts described above were combined to obtain an experimental design with four different scenarios. More precisely, Scenario 1 compared private economy options to private health options, Scenario 2 compared private economy options to public health options, Scenario 3 compared public economy to private health options, and Scenario 4 compared public economy to public health options.

From the 16 different options, a number of $$\left( {\begin{array}{c}16\\ 2\end{array}}\right) =120$$ stimuli for each of the four scenarios was obtained. To give an example, one of the stimuli of Scenario 2 comparing private economy to public health options is the following one:

*The government has established new COVID-19 regulations that may have the following consequences. Which alternatives do you prefer? *

1. *Your family income decreases by 5% and there are 200 more deaths from COVID-19 than yesterday.*
2. *Your family income remains unchanged and there are 500 more deaths from COVID-19 than yesterday.*

In all scenarios, each stimulus consists of a pair $\{(e_1,h_1),(e_2,h_2)\}$ of options, where $e_1,e_2 \in E_i$ and $h_1,h_2 \in H_i$. For convention, the former element of each option refers to the economic component, while the latter refers to the health component.

In the context of the present application, the higher the level, the higher the severity of the situation described by the level. Thus, in a stimulus $\{(e_1,h_2),(e_2,h_3)\}$, if Condition (4) holds, then $(e_1,h_2)$ is preferred over $(e_2,h_3)$. This assumption is in line with the so-called monotonicity axiom.

In each of the four scenarios, the total number of monotone stimuli is 84. The remaining stimuli (i.e., a number of 36) are non-monotone (see “Data sets generation and simulation design” section for the definitions of monotone and non-monotone stimuli). Thus, the total number of monotone and non-monotone stimuli considering all the four scenarios would be $120 \times 4=480$. Given such a huge number of stimuli, the data collection was split into two studies, each of which had a different aim. In the former study, the aim was to verify if the monotonicity axiom holds in the context of a lockdown social dilemma. In the latter, the aims were those presented in “Aims and scopes” section and the study was carried out considering only the non-monotone stimuli. Thus, in Study 1, participants answered to all the 120 monotone and non-monotone stimuli of a single scenario. In Study 2, participants answered to only the non-monotone stimuli of all four scenarios, which is a number of $4 \times 36=144$ stimuli. In the two studies, the data were collected by using two different samples. A detailed explanation of these studies is given in the subsequent two sections.

Regarding the tools used for collecting the data, two questionnaires, one for each study, were developed by using the software Qualtrics (https://www.qualtrics.com), an online platform that allows to collect and manage experiential data via the World Wide Web. Each of the two anonymous questionnaires comprised different blocks. The first block explained the type of task the respondent would expect and some stimulus examples. A second block asked for the approval of the informed consent. A third block asked for some socio-demographic information (gender, age, instruction level) of participants. Finally, the last block comprised the experimental stimuli that were administered in random order. Of course, participants were free to exit the questionnaire at any time.

The material used in the present empirical application and in the simulation study is publicly available at the Open Science Framework url: https://osf.io/gsdvn/?view_only=b99ccb676d3d4e2bb3e7a3310c4be76f.

The material consists of links to the Qualtrics questionnaires used in the empirical studies, MATLAB functions used in both the simulations and the empirical studies, and the mat files of all the data sets used in the empirical studies.

Regarding the speed of convergence of the applied algorithms, across 600 replications, the average convergence times were $2.8 \times 10^{-3}$ s ($SD = 0.001$), $2.9 \times 10^{-2}$ s ($SD= 0.017$), and $1.0 \times 10^{-1}$ s ($SD = 0.058$) for recovering preference structures with 10, 50, and 100 states, respectively. For a more detailed analysis of convergence performance under varying error conditions, the reader is referred to the supplementary material. All simulations were performed on a Dell XPS equipped with a 12th Gen Intel Core i7-1260P processor (2.10 GHz) and 16 GB of RAM.

### Study 1: Violation analysis

#### Participants

A total of 154 adults with an average age of 41.5 years (*SD* = 18.5 years) were recruited for the study using the snowball sampling method. No compensation was provided for participation. Of the participants, 53% were female, 29% were male, and the remainder did not respond to the gender question. The questionnaire was administered online and was completed in a median time of 15 min.

A total of 26.5% of participants did not consent to the processing and dissemination of their data and were therefore excluded from the study, with no data collected from them. An additional 31% did not respond to any of the stimuli. The remaining 66 participants (43%) responded to at least one stimulus and formed the final dataset. These participants had an average age of 42.8 years (*SD* = 19.0), and 61% were female. Among them, 54 responded to all stimuli, while the remaining 12 had at least one missing response.

An important percentage of the participants providing at least one missing answer was not unexpected and could be attributed to two factors. First, the substantial number of stimuli (i.e., 120) might have contributed to the fatigue or disengagement. Second, the difficulty of FCPC in some circumstances may have led participants to skip stimuli instead of attempting to answer.

#### Methods

The first step consisted of selecting the data of the 84 monotone-stimuli only. Each was coded as 1 if the observed response violated the monotonicity axiom, and as 0 otherwise. The plausibility of the assumption was verified by computing the probability of responding correctly to a monotone stimulus. This probability was estimated from the data by maximum likelihood, computing the average value

$$ \hat{p}=1-\frac{1}{N} \sum _{s=1}^N p_s, $$

where $p_s$ is the proportion of monotone stimuli in which a violation of Condition (4) was observed for subject *s*.

#### Results and discussion

Only the results obtained for Scenario 2 (private economy vs. public health) are described here. Very similar results were obtained for the other scenarios.

The proportion $p_s$ of monotone stimuli for which a violation of Condition (4) was observed ranged from a minimum of 0 to a maximum of .55. This last proportion was observed in only one participant, who was excluded from subsequent data analyses. In fact, their choices were systematically for the worse condition (e.g., more dead rather than less dead, or less money rather than more money). Instead, most of the participants (i.e., 42 out of 66) never violated (MA) – the median number of violations was zero. The estimated probability of responding coherently with the monotonicity axiom was $\hat{p}=.98$, providing strong evidence in favor of that axiom. For this reason, Study 2 was designed only on the non-monotone stimuli.

It is worth mentioning some limitations of the study concerning generalizability and reliability. Specifically, over half of the participants were removed from the sample because they did not complete all 120 stimuli. It is important to investigate the missing data mechanisms to determine whether the missing data were random or systematic. However, given the illustrative purpose of the study, this aspect does not undermine the study’s primary contributions.

### Study 2

#### Participants

The sample consisted of 555 adults (53.6% female, 14.2% male, and 32.2% non-disclosed) with an average age of 23.4 years ($SD = 6.8$). Most were psychology students at the University of Padua. The questionnaire was administered online and it was completed, on average, in about 20 min. Participants were recruited using the snowball sampling method and no compensation was provided for their participation.

Forty-eight participants did not provide informed consent for the processing and dissemination of their data, so their data were not recorded. Of the remaining 507 participants, 110 did not complete the experiment. Additionally, participants with any missing data in a given scenario were excluded from that scenario, as the clustering algorithms require complete data. The final sample comprised 378 participants in the private economy vs. private health scenario, 376 participants in the private economy vs. public health scenario, 383 participants in the public economy vs. private health scenario, and 375 participants in the public economy vs. public health scenario. Like in Study 1, the high rate of participants providing at least one missing answer was not unexpected for the same reasons mentioned above.

#### Methods

In each scenario, a two-step procedure was applied in which: (1) two preference structures were extracted from the data, one by using *k*-orders with the TCA procedure and the other by using *k*-modes; and (2) the two extracted preference structures were tested empirically by fitting a probabilistic model, named basic local independence model (Falmagne & Doignon, 1988a, b), to the data. The BLIM based on the structure extracted by *k*-modes was then compared with the BLIM based on the structure extracted by *k*-orders, via the Akaike information criterion (AIC) and its corrected version (AICc). These two steps are described in detail below.

The extraction of the preference structures (Step 1), was performed following cross-validation techniques. The sample of each scenario was randomly partitioned into two independent sets. One of the two sets, named training set $\mathcal {T}$, comprised two-thirds of the sample and was used for extracting the structure. The other set, named validation set $\mathcal {V}$, comprised the remaining sample and was used for testing the extracted structure. One hundred different partitions were generated and for each of them, 96 conditions were considered in which the cardinality of the initial set of states on which the algorithms started varied in the interval $\{4, 5, \cdots , 100\}$. In each partition *i* and in each condition *j* of every scenario, the two algorithms *k*-modes and *k*-orders were applied to the training set $\mathcal {T}_{ij}$ for extracting the preference structures.

In each scenario, among the 96 (conditions) $\times 100$ (partitions) $\times 2$ (algorithms) $=$ 19,200 extracted structures, two “best structures” $\mathcal {P}_{k\text {-orders}}$ and $\mathcal {P}_{k\text {-modes}}$ were selected. For each of the two algorithms, the procedure proposed by de Chiusole et al. (2020) was used for constructing a selection criterion. For each validation set $\mathcal {V}_i$ and each preference structure $\mathcal {P}_{ij}$, first the two distances $d_1=d(\mathcal {V}_i,\mathcal {P}_{ij})$ and $d_2=d(\mathcal {P}_{ij},\mathcal {V}_i)$ were computed by using Equation (3). Then, the maximum $M_{ij}=\text {max} \{d_1,d_2\}$ of the two distances was taken for each condition *j* of each partition *i*. The best-extracted structure was the one having the smallest $M_{ij}$. In this way, the two best-extracted structures were obtained, one for each algorithm. A more detailed description of all passages for computing the selection index can be found in de Chiusole et al. (2020).

Concerning Step (2), it consisted of the application of the basic local independence model (BLIM; Falmagne & Doignon, 1988a; Heller & Wickelmaier, 2013; Stefanutti & Robusto, 2009), based on the preference structures extracted in Step (1), to the data. The BLIM is one of the most used probabilistic models in knowledge space theory (KST; Doignon & Falmagne, 1999; Falmagne & Doignon, 2011; Falmagne et al., 2013; Heller & Stefanutti, 2024) for empirically validating knowledge structures.

Knowledge structures represent the knowledge organization in a particular domain. They are formed by a nonempty collection of knowledge states, that is, subsets of items that a student is able to solve. If a population of students is considered, then all the knowledge states existing in the population form the knowledge structure $\mathcal {K}$. It is plausible to assume the existence of a probability distribution $\pi _{\mathcal {K}}$ on the collection of states $K \in \mathcal {K}$.

A knowledge state $K \in \mathcal {K}$ cannot be directly observed. What is observed is the response pattern of a student, that is, the subset $X \subseteq Q$ of problems correctly solved by the student. The perfect identity between *K* and *X* might not exist due to random error (noise in the data). Thus, the relationship between the two is established by a restricted latent class model, where the states $K \in \mathcal {K}$ are the latent classes. In this model, the probability $\mathbb {P}(X)$ of observing *X* in a randomly sampled student is computed by

5 $$\begin{aligned} \mathbb {P}(X) = \sum _{K \in \mathcal {K}} \mathbb {P}(X|K) \pi _K, \end{aligned}$$

where $\mathbb {P}(X|K)$ is the conditional probability of observing the response pattern *X* given that the knowledge state of the student is *K* and $\pi _K$ is the probability of *K*. Under the assumption of local independence of the item responses given the knowledge states, the conditional probability $\mathbb {P}(X|K)$ takes on the form:

6 $$\begin{aligned} \mathbb {P}(X|K)=\left[ \prod _{q \in K \setminus X} \beta _q\right] \left[ \prod _{q \in K \cap X} (1-\beta _q)\right] \left[ \prod _{q \in X \setminus K} \eta _q\right] \left[ \prod _{q \in Q \setminus (X \cup K)} (1-\eta _q)\right] , \end{aligned}$$

where $\beta _q$ is the conditional probability of observing an incorrect answer to an item $q \in Q$ given that $q \in K$, and $\eta _q$ is the conditional probability of observing a correct response to an item $q \in Q$, given that $q \notin K$. These two parameters represent, respectively, the situation of false negatives and false positives.

Several theoretical and empirical applications of the model exist in the literature. A brief list is here reported: de Chiusole et al. (2013, 2015); Anselmi et al. (2016); Heller (2017); de Chiusole et al. (2013); Heller and Wickelmaier (2013); de Chiusole et al. (2020); Anselmi et al. (2021). The reader is referred to this literature for more details on this model.

For applying the BLIM to the preference structures extracted in Step (1), a correspondence between the relevant concepts in KST and those used in this application was established and applied. The following example shows how Equation (6) is applied in the case of a simple set $S=\{a,b,c\}$ of options. There are on the whole eight different preference relations (see Table 1). Each of them is an observable response pattern. Suppose that the clustering algorithm extracted the following four centroids:

$$\begin{aligned} \{(a,b),(b,c),(a,c)\}\\ \{(a,b),(c,b),(c,a)\}\\ \{(a,b),(c,b),(a,c)\}\\ \{(b,a),(b,c),(a,c)\} \end{aligned}$$

For applying Equation (6), the error parameters $\beta$ and $\eta$ are indexed by pairs of options in *S*, obtaining the following six parameters: $\beta _{(a,b)}$, $\beta _{(a,c)}$, $\beta _{(b,c)}$, $\eta _{(a,b)}$, $\eta _{(a,c)}$, and $\eta _{(b,c)}$. For example, the parameter $\beta _{(a,b)}$ is interpreter as the probability that the pair (*b*, *a*) belongs to the observed response pattern given that the pair (*a*, *b*) belongs to the preference state. Conversely, the parameter $\eta _{(a,b)}$ is interpreter as the probability that the pair (*a*, *b*) belongs to the observed response pattern given that (*b*, *a*) belongs to the preference state. It should be observed that for every pair of options $(x,y) \in S \times S$, the equality $\beta _{(x,y)}=\eta _{(y,x)}$ holds true. Given this definitions, an application of (6) to $R=\{(a,b),(b,c),(c,a)\}$ and $P=\{(b,a),(b,c),(a,c)\}$ gives:

$$ \mathbb {P}(R|P)=\beta _{(b,a)} \cdot (1 - \beta _{(b,c)}) \cdot \beta _{(a,c)}. $$

Then, Step (2) was carried out by applying the BLIM to the data of each scenario by using both the best preference structures $\mathcal {P}_{k\text {-orders}}$ and $\mathcal {P}_{k\text {-modes}}$ extracted by the corresponding algorithm. The model parameters were estimated by maximum likelihood via the expectation-maximization algorithm, adapted for the BLIM by Stefanutti and Robusto (2009). The goodness-of-fit of the model was tested by using the Pearson Chi-square statistic, whose *p* value was obtained by a parametric bootstrap of over 1000 replications. The identifiability of the model could not be tested by using formal methods, like, e.g., the BLIMIT (Stefanutti et al., 2012). This method requires building the entire Jacobian matrix of the model’s prediction function. The number of rows of this matrix equals the number of theoretically observable response patterns, which, in the case at issue, is $2^{36}$, an unreachable number. Nevertheless, an empirical procedure (Stefanutti et al., 2020, 2021) was used that did not find any identifiability issue of the model parameters of both the best preference structures $\mathcal {P}_{k\text {-orders}}$ and $\mathcal {P}_{k\text {-modes}}$.

Finally, as an example of the practical usefulness of the procedure, some of the most interesting results about the extracted preference structures are presented only for *k*-orders. In all scenarios, each state of preference $P \in \mathcal {P}_{k\text {-orders}}$ can be partitioned into two subsets. The former subset contains all those stimuli for which a preference for the option having the least level of severity for the health component was exhibited, that is

$$ P_{H_c}=\Big \{ \{(e_i,h_i),(e_j,h_j)\} \in P : h_i \prec h_j \wedge e_j \prec e_i\Big \}, $$

where $e_i,e_j \in E_c$ and $h_i,h_j \in H_c$. The latter subset contains all those stimuli for which a preference for the option having the least level of severity for the health component was exhibited, that is

$$ P_{E_c}=\Big \{ \{(e_i,h_i),(e_j,h_j)\} \in P : h_j \prec h_i \wedge e_i \prec e_j \Big \}. $$

Of course, $$P_c=P_{H_c} \cup P_{E_c}$$.

With the aim of comparing, in each scenario, the estimated probability of preferring one of the two components, two disjoint collections of states can be formed from the preference structure $\mathcal {P}_{k\text {-orders}}$. The former is the collection of all preference states for which a higher weight is given to the economic component, that is

$$ \mathcal {P}_E=\{P \in \mathcal {P}_{k\text {-orders}}: |P_{E_c}| >|P_{H_c}|\}, $$

where $|P_{E_c}|$ and $|P_{H_c}|$ are the cardinalities of the two subsets $P_{E_c}$ and $P_{H_c}$, defined above.

The latter is the collection of preference states for which a higher weight is given to the health component, that is

$$ \mathcal {P}_{H_c}=\{P \in \mathcal {P}_{k\text {-orders}}: |P_{H_c}| >|P_{E_c}|\}. $$

Another useful aspect of a preference state is its occurrence probability $\pi _P$ that can give information about the relevance of a state in the reference population. Two aggregate indexes based on the occurrence probabilities of the states belonging to one or the other collection $\mathcal {P}_{E_c}$ and $\mathcal {P}_{H_c}$ can be computed. The former is the probability of preferring the health component that is computed by

$$ \mathbb {P}_{H_c}= \sum _{P \in \mathcal {P}_{H_c}} \pi _P, $$

where $\pi _P$ is the probability estimated by the BLIM for the state *P*. The latter probability is the probability of preferring the economic component that is computed by

$$ \mathbb {P}_{E_c}= \sum _{P \in \mathcal {P}_{E_c}} \pi _P. $$

Additionally, graphical representations of preference structures are developed and displayed, which may facilitate the interpretation of the results. An *(undirected) graph* for the preference structure $(A,\mathcal {P})$ is a pair (*V*, *E*) of nonempty sets, where *V* is a set of *graph vertices* bijectively corresponding to the binary relations in $\mathcal {P}$, and $E \subseteq V \times V$ is a set of the *graph edges*. Specifically, a pair (*u*, *v*) of vertices in *V* is a graph edge (i.e., $(u,v) \in E$) if and only if the binary relations represented by *u* and *v* are neighbors in the sense specified in “Preliminary definitions and theoretical results” section.

**Fig. 3:**
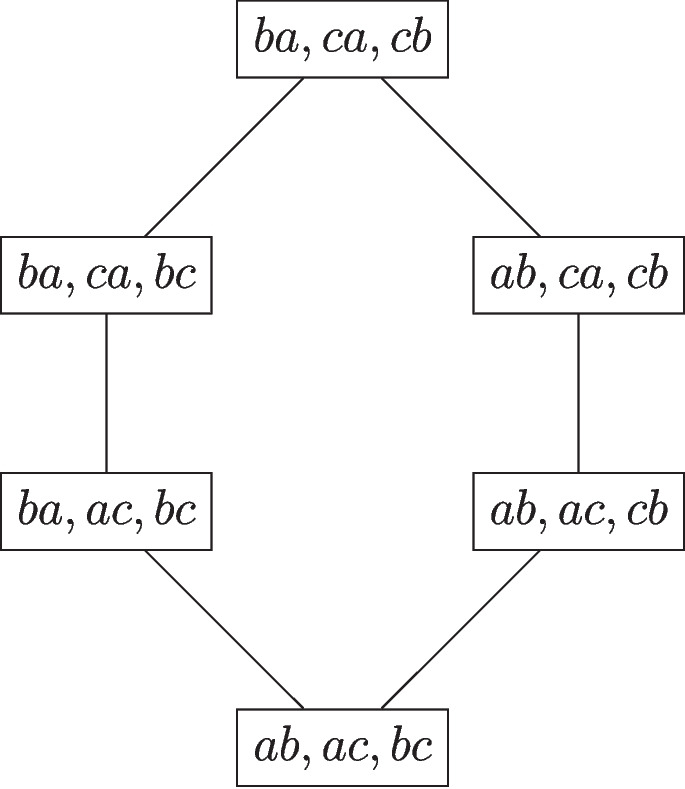
Graph of the preference structure consisting of all the transitive relations on a set of three options

As an illustrative example, consider again the eight preference relations listed in Table 1, and suppose that $\mathcal {P}$ contains the six that are transitive. Then the graph of $\mathcal {P}$ is the one displayed in Fig. 3.

#### Results

Table 5 summarizes the absolute and relative fit indexes obtained when the BLIM was applied to the data by using $\mathcal {P}_{k\text {-orders}}$ (Columns 2 to 4) and $\mathcal {P}_{k\text {-modes}}$ (Columns 5 to 7).

In all scenarios, the BLIM fitted the data pretty well with both structures. The comparison between the BLIM fitted by using $\mathcal {P}_{k\text {-orders}}$ and that fitted by using $\mathcal {P}_{k\text {-modes}}$ do not show a clear superiority of one model against the other. In fact, in Scenarios 1 and 3, the BLIM based on $\mathcal {P}_{k\text {-modes}}$ obtained the smallest AIC and AICc, whereas in Scenarios 2 and 4 the BLIM based on $\mathcal {P}_{k\text {-orders}}$ obtained the smallest values. Nevertheless, the differences among the comparison fit indexes of the two models are negligible, in all scenarios. Considering that *k*-orders is a constrained version of *k*-modes, one would expect the best performance of the latter. Thus, these results provide mild support to the hypothesis that in the social context here considered, preference is transitive.

**Table 5:** Absolute and relative fit indexes obtained, in Study 2 of the empirical application, for the BLIM when it is applied to the data of each scenario (rows of the table) by using the preference structure extracted by *k*-orders (Columns 2 to 4) and by *k*-modes (Columns 5 to 7)

	*k*-orders			*k*-modes		
Scenario	*p* value	AIC	AICc	*p* value	AIC	AICc
1	1.000	2261	2,570	.9982	2,112	2,334
2	1.000	2657	2944	.9980	2734	3008
3	1.000	2833	3135	1.000	2822	3124
4	1.000	3822	4498	1.000	3853	4177

In the subsequent paragraphs, some results on the parameter estimates of the BLIM and on the extracted preference structure are given only for the *k*-orders algorithm. The reason is that the outcome of the *k*-modes algorithm may not be interpretable, as the cluster centroids are not necessarily valid preference orders.

**Fig. 4:**
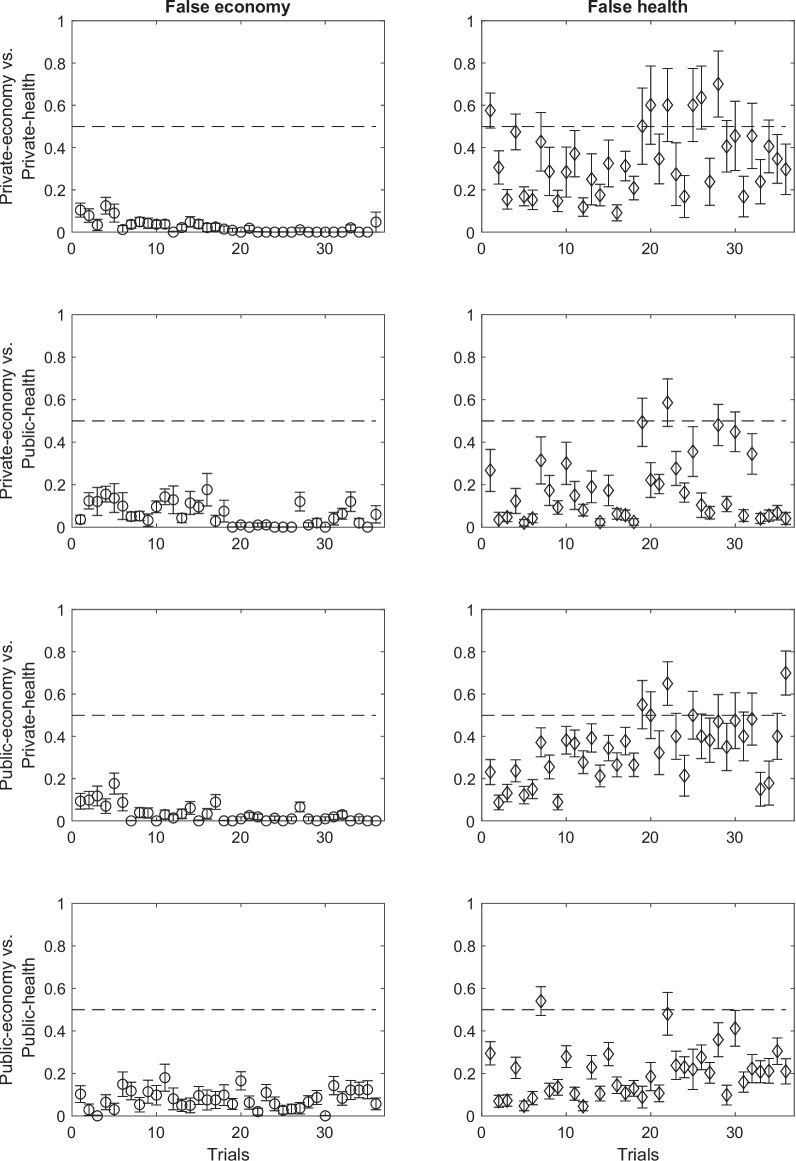
False economy and false health parameter estimates obtained in Study 2 of the empirical application

Figure 4 shows the estimated “false economy” (panels on the left) and “false health” (panels on the right) probabilities of the BLIM, for each of the four scenarios (row panels in the figure). In each panel, the stimuli are along *x*-axis.

False economy probability refers to the likelihood that a subject, who has a preference for the health component of a stimulus, responds with a preference for the economy component. Conversely, false health probability is the likelihood that a subject, who prefers the economy component, responds with a preference for the health component. Interestingly, the figure clearly shows that false health probabilities are almost consistently higher than false economy probabilities across all scenarios.

This result can be interpreted by the social-desirability bias (Edwards, 1953). Indeed, preferring health against the economy could be seen as an expected answer that conforms with a “good behavior” of a citizen. Table 6 summarizes the average values of these estimates (Columns 3 and 4).

**Table 6:** Cardinality of the preference structures and average values of parameter estimates in the four scenarios of Study 2 of the empirical application

Scenario	$|\mathcal {P}_{k\text {-orders}}|$	$\bar{\beta }_q$ (SD)	$\bar{\eta }_q$ (SD)
1	10	.03 (.03)	.34 (.17)
2	8	.06 (.05)	.17 (.15)
3	10	.03 (.04)	.34 (.15)
4	24	.08 (.04)	.20 (.12)

On average, false health $\hat{\eta }_q$ probabilities are greater than false economy $\hat{\beta }_q$ probabilities, especially in “private economy vs. private health” and “public economy vs. private health” scenarios. These two scenarios have in common the “private health” context. Thus, it seems that when private health is considered, some subjects answered with a preference for health even when, actually, they preferred the economy component.

**Fig. 5:**
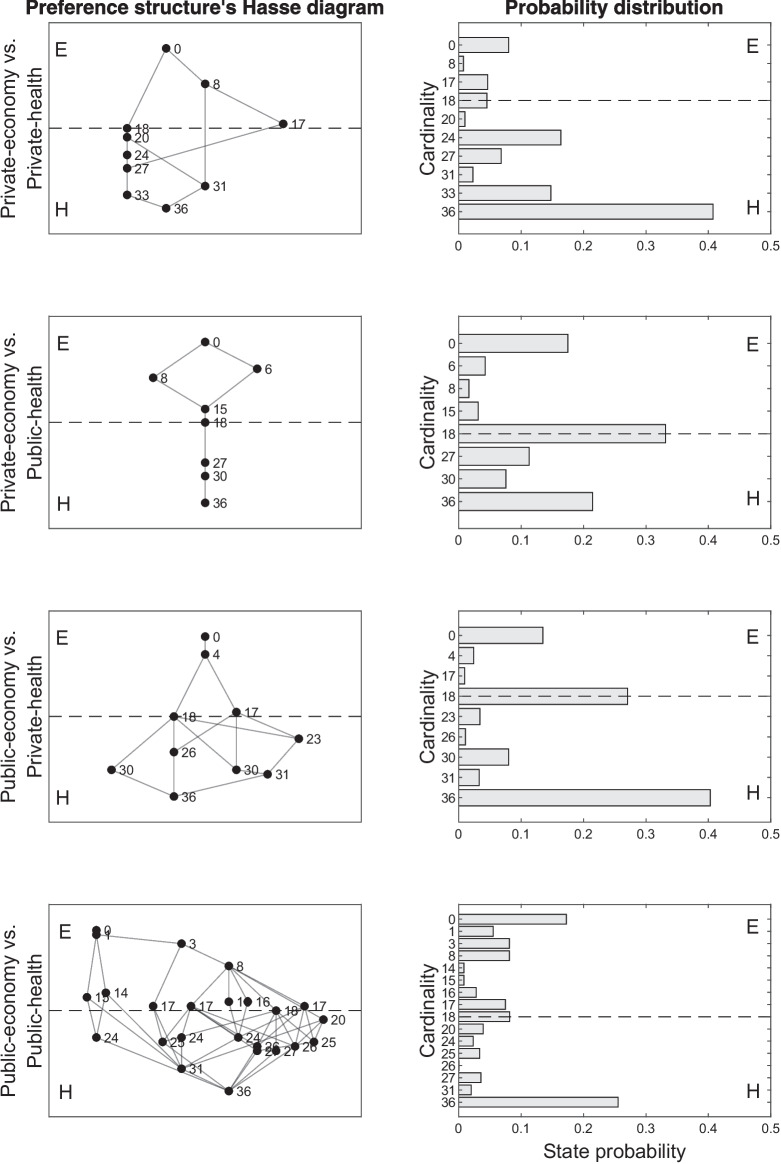
Graphs of the preference structures extracted by *k*-orders (*left panels*) and the corresponding probability distributions (*right panels*), in the four scenarios (*row panels*) of Study 2. See text for more details

Figure 5 shows the graph of the preference structures extracted by *k*-orders (panels on the left) and the corresponding probability distribution among the preference states (panels on the right). Row panels refer to the four scenarios. In each panel on the left, a dashed line splits the Cartesian plane into two areas. The upper areas contain all the states belonging to $\mathcal {P}_{E_c}$, that is, those preference states giving higher weight to the economic component. Whereas the lower areas contain all the states belonging to $\mathcal {P}_{H_c}$, that is those preference states giving higher weight to the health component. The states that fall exactly on the dashed lines give equal weight to the two components. Each node of the graph represents a preference state $P \in \mathcal {P}_{k\text {-orders}}$ and a number close to the node is the cardinality of $P_{H_c}$ (number of stimuli for which a preference for the option having the least level of severity for the health component was exhibited).

It can be seen from the figure that, in general, the number of states belonging to $\mathcal {P}_{H_c}$ (area below the dashed line) is greater than the number of states belonging to $\mathcal {P}_{E_c}$ (area above the dashed line). The only exception was for the private economy vs. public health scenario. This is a first suggestion of the fact that, in general, the subjects of the sample gave higher weight to the health component, in almost all scenarios. A similar result should also take into account the probability distribution on the states.

Panels on the right of Fig. 5 display the probability distribution of the preference states represented in the corresponding graph. The estimated probability of the states is along the *x*-axis, whereas cardinalities of the states, sorted from the smallest to the highest, are along the *y*-axis. The dashed line is used here exactly like in the left panels. Several interesting results arise.

**Fig. 6:**
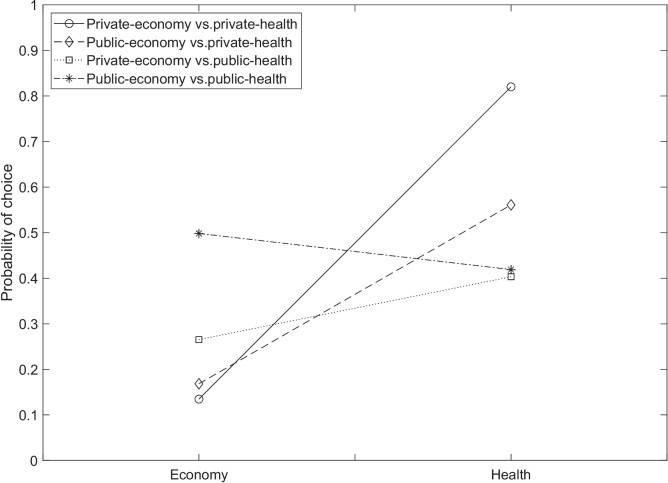
Comparison between probability of choosing the economic component and that of choosing the health component in the four scenarios of the Study 2 of the empirical application. More details are given in the text

When the private economy is compared with the private health (Scenario 1), about 40% of the sample has a preference state whose cardinality is equal to $|P_{H_c}|=36$. The only state having 36 as cardinality is the state in which the option with the least severity for health was chosen in all stimuli. This state represents the situation of a strong preference for health. On the other hand, less than 10% of the sample has a preference state whose cardinality is equal to $|P_{H_c}|=0$. The only state having zero as cardinality is the state in which the option with less severity for the economy was chosen in all stimuli. This state represents the situation of a strong preference for the economy. Other quite interesting states are those having $|P_{H_c}|=18$ as cardinality, which are states giving equal weight to the two components. In this scenario, the percentage of the sample having such states is very small (i.e., about 5%). Concerning the remaining preference states, it can be noted that the mass of the distribution falls mostly in the health area of the panel. Among the four scenarios, this one seems to be the one for which the biggest agreement among subjects exists in preferring health against the economy.

When the private economy is compared with public health (Scenario 2), the percentage of subjects that give equal weight to the two components drastically increases to more than 30%. On the other hand, the percentage of subjects having a strong preference for the health component is halved and similar to that of the subjects having a strong preference for the economy component (i.e., about 20%). Concerning the remaining states, those falling in the health area have a slightly larger probability than the states in the economic area.

When the public economy is compared with private health (Scenario 3), most part of the sample (i.e., about 40%) has a strong preference for health, similarly to what observed in Scenario 1. Moreover, similarly to Scenario 2, a quite large percentage of subjects (about 30%) gives equal weight to the two components. Interestingly enough, Scenarios 2 and 3 are those in which a social dilemma exists (i.e., one of the two components refers to a private context and the other to a public context). It does not come as a surprise that in these two scenarios, the percentages of subjects that gave equal weight to the two components are greater than those of the other two scenarios, where it is less than 10%.

Finally, when the public economy is compared with public health (Scenario 4), the probability mass is distributed on a greater number of states. Even if a strong preference for the health component is observed for about 25% of the sample, the mass of the distribution on the other states seems to be higher in the economic area.

For summarizing the results obtained in the four scenarios, the probability $\mathbb {P}_{E_c}$ of all those states that give greater weight to the economic component was compared to the probability $\mathbb {P}_{H_c}$ of the states that give greater weight to the health component. Figure 6 shows these probabilities for each scenario. The two components are along the *x*-axis, whereas the probabilities $\mathbb {P}_{H_c}$ and $\mathbb {P}_{E_c}$ are along the *y*-axis. Each curve represents one of the four scenarios, as explained by the legend.

It is quite evident that in three scenarios out of four, the probability $\mathbb {P}_{H_c}$ of the health component is higher than the probability $\mathbb {P}_{E_c}$ of the economic component. The only scenario in which the opposite trend was observed was Scenario 4 (in which public context was considered for both components). Interestingly enough, in Scenario 1 (private economy vs. private health) the economic component probability is the lowest, whereas the probability of the health component is the highest. On the other hand, in Scenario 4 (public economy vs. public health) the probability of the economic component is the highest, whereas the probability of the health component is one of the lowest. Thus, it appears that when subjects must give a preference for health or for economy components, they answer in an opposite way, depending on the private or public context in which both components are described. In the private context, they seem to give greater weight to health in the public context to economy.

In the two mixed-context scenarios (2 and 4), the probability of the health component is always the highest, regardless of the public or private context in which it is defined. Nevertheless, the difference between the two probabilities $\mathbb {P}_{H_c}$ and $\mathbb {P}_{E_c}$ is about four times lower in the private economy vs. public health scenario (i.e., .12 against .45). Also these last results could be interpreted in the light of the social dilemma. More precisely, the data suggest that the preference of preserving health against the economy is particularly strong when health refers to the private context and economy refers to the public context. However, this preference decreases a lot when health refers to the public context and economy refers to the private context. Thus, it appears that the social dilemma acts as a moderator of individual preference between health and economy, rather than fully determining that preference.

#### Discussion

Results of the study demonstrated the real-world application of the procedure, revealing heterogeneous preference states among participants and challenging the assumption of uniform preferences. The *k*-order and *k*-modes algorithms effectively captured these variations, supporting the hypothesis that preferences cannot be homogeneous in a population. When comparing the performances of *k*-order to those of *k*-modes, neither model showed clear superiority, both fitting the data well with minor differences. However, considering that *k*-orders is a constrained version of *k*-modes, one would expect the best performance of the latter. This suggests that in the social context examined, preferences can be considered transitive. Overall, these findings highlight the effectiveness of the algorithm in capturing diverse preference states and underscore the complexity of real-world individual preferences.

Concerning the context of the lockdown social dilemma, the following results were obtained. First, the results indicate that there is a notable discrepancy between stated preferences and actual preferences regarding health and economy components in stimuli. Specifically, the probability of an individual falsely indicating a preference for health is consistently higher than the probability of falsely indicating a preference for the economy. These results could suggest a tendency among participants to conform to socially desirable responses, especially when it comes to prioritizing health over the economy, highlighting the influence of social norms on decision-making processes.

Second, the number of preference states favoring the health component is generally greater than the number of states favoring the economy component. The only exception to this trend was observed in the “private economy vs. public health” scenario, where preference for economy is higher. These observations suggest two key points: (1) The sample tended to prioritize health over the economy in most scenarios, indicating a general preference for health-related decisions. However, this is not the case when public health interests are compared to private economy interests; (2) Participants who preferred health exhibited more heterogeneity in their preferences (i.e., the number of preference states is higher) compared to those who preferred the economy. This heterogeneity implies that while many participants lean towards prioritizing health, their preferences and the reasons behind them vary more widely than those who prioritize economy.

In conclusion, thanks to the *k*-order algorithm, it was possible to analyze the diversity in health-related preferences within a complex decision-making context, such as the lockdown social dilemma.

Nevertheless, some limitations of the study regarding its generalizability and reliability need to be clarified. Besides the issues concerning missing responses (discussed in Study 1), it is noteworthy that the demographics of the samples in Study 1 and Study 2 differ substantially. Study 1 comprised predominantly female participants who were, on average, older than participants in Study 2. Since the results of Study 1 were used to focus exclusively on non-monotone stimuli in Study 2, the demographic disparity may indicate potential issues of reliability and limited generalizability of the study. Addressing these aspects could strengthen the conclusions and might offer a more comprehensive understanding of the study’s applicability. However, considering the illustrative purpose of the studies, these weaknesses do not undermine their primary contribution of this application.

## Summary and final remarks

A clustering algorithm, named *k*-orders, was proposed to extract asymmetric, complete, and transitive binary relations from preexisting data sets.

The *k*-orders algorithm differs from the original *k*-modes in the adjustment step, while the classification step remains the same. Two adjustment procedures named *transitive centroid adjustment* (TCA) and greedy TCA were proposed whose aim is to find a strict linear order (transitive, asymmetric, and complete binary relation) which is at a minimum distance from the cluster.

The difference between the two procedures lies in their approach to constructing order relations. The TCA algorithm starts from an arbitrary strict linear order and iteratively modifies it to minimize the distance from the cluster. In contrast, the greedy TCA algorithm begins with an empty set and constructs a specific order relation by adding one pair of options at a time, following a pre-specified sequence, until no further pairs can be added. It is considered a “greedy” algorithm because it evaluates only one step ahead at each stage.

The set of cluster centroids extracted by the algorithm from a data set is then empirically tested by applying a constrained latent class model, where the latent classes are the extracted centroids, to another data set. The model used in the present research was the BLIM. This model was chosen among other options (e.g., BTL with latent classes or random utility models) because it makes no assumptions about the measurement level of preference but it is restrictive enough for testing whether an assumption like transitivity holds true in the data or not. The good fit of the model to the data favors the assumption that centroids are transitive.

The performance of the two versions of *k*-orders were compared to one another and with the canonical *k*-modes, in simulation studies. Results show that when centroids are transitive relations, both versions of *k*-orders outperform *k*-modes. Moreover, in experimental designs in which two-component options are considered, TCA algorithm performs better than the greedy TCA.

An empirical application was also carried out for exemplifying how *k*-orders can be useful for studying individual preferences. In particular, preference structures were extracted from data collected during the Coronavirus (COVID-19) pandemic. The aim was to study the individual preferences in different scenarios involving or not a “lockdown social dilemma”, comparing the *economy* and the *health* components. Several interesting results were obtained and discussed in “Results” section. Here, it is only underlined that the approach was useful for identifying different preference orders whose interpretation can be used for answering specific research questions on the nature of the individual preferences.

The proposed algorithms also have some limitations that have to be mentioned. Indeed, neither the TCA nor the greedy TCA procedures can assure a globally optimal solution (see Propositions 2 and 4). The problem of finding a global optimum is a well-known issue in *k*-modes clustering algorithms (Chaturvedi et al., 2001). However, the positive results obtained in the simulation study of “Simulation Study 3: The selection criterion” section might suggest that the performance of the two TCA procedures is excellent in comparison to *k*-modes, and this happens irrespectively of the either local or global solution found. The global minimum issue could be (at least) managed by using supplementary versions of the algorithms, which also perform an initial exploration of the solution space in order to reduce the probability of incurring in a local minimum. de Chiusole et al. (2017) proposed the *k*-states algorithm which is an incremental extension of the canonical *k*-modes, designed to generate a sequence of progressively larger knowledge structures, from which the “best” solution is selected. A similar approach could potentially be applied to the algorithm proposed in this study. It is reasonable to expect that the introduced constraints-such as transitivity and the Monotonicity Axiom-reduce the number of feasible solutions, thereby decreasing the number of local minima and increasing the likelihood of reaching the global minimum. While this aspect is promising, it lies beyond the scope of the current study and could serve as a direction for future research.

## Open-practices statement

The data and the supplementary materials are available at the following link: https://osf.io/gsdvn/?view_only=b99ccb676d3d4e2bb3e7a3310c4be76f. None of the experiments was preregistered.

## Data Availability

The data and the supplementary materials are available at the following link: https://osf.io/gsdvn/?view_only=b99ccb676d3d4e2bb3e7a3310c4be76f.

## Footnotes

1 The supplementary material is available at https://osf.io/gsdvn/?view_only=b99ccb676d3d4e2bb3e7a3310c4be76f.

2 The supplementary material is available at https://osf.io/gsdvn/?view_only=b99ccb676d3d4e2bb3e7a3310c4be76f.

## Code availability

The code is available at the following link: https://osf.io/gsdvn/?view_only=b99ccb676d3d4e2bb3e7a3310c4be76f.
